# Coupled Natural Fusion Enzymes in a Novel Biocatalytic Cascade Convert Fatty Acids to Amines

**DOI:** 10.1021/acscatal.2c02954

**Published:** 2022-10-05

**Authors:** Shona M. Richardson, Piera M. Marchetti, Michael A. Herrera, Dominic J. Campopiano

**Affiliations:** School of Chemistry, The University of Edinburgh, David Brewster Road, EdinburghEH9 3FJ, U.K.

**Keywords:** biocatalysis, cascade, pyridoxal 5′-phosphate, thioester reductase, transaminase, tambjamine biosynthesis

## Abstract

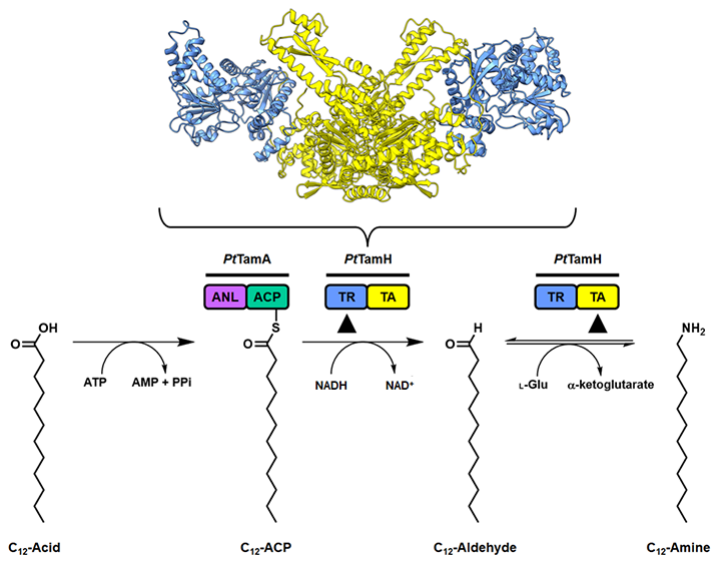

Tambjamine YP1 is a pyrrole-containing natural product. Analysis of the enzymes encoded in the *Pseudoalteromonas tunicata* “*tam*” biosynthetic gene cluster (BGC) identified a unique di-domain biocatalyst (*Pt*TamH). Sequence and bioinformatic analysis predicts that *Pt*TamH comprises an N-terminal, pyridoxal 5′-phosphate (PLP)-dependent transaminase (TA) domain fused to a NADH-dependent C-terminal thioester reductase (TR) domain. Spectroscopic and chemical analysis revealed that the TA domain binds PLP, utilizes l-Glu as an amine donor, accepts a range of fatty aldehydes (C_7_–C_14_ with a preference for C_12_), and produces the corresponding amines. The previously characterized *Pt*TamA from the “tam” BGC is an ATP-dependent, di-domain enzyme comprising a class I adenylation domain fused to an acyl carrier protein (ACP). Since recombinant *Pt*TamA catalyzes the activation and thioesterification of C_12_ acid to the *holo*-ACP domain, we hypothesized that C_12_ ACP is the natural substrate for *Pt*TamH. *Pt*TamA and *Pt*TamH were successfully coupled together in a biocatalytic cascade that converts fatty acids (FAs) to amines in one pot. Moreover, a structural model of *Pt*TamH provides insights into how the TA and TR domains are organized. This work not only characterizes the formation of the tambjamine YP1 tail but also suggests that *Pt*TamA and *Pt*TamH could be useful biocatalysts for FA to amine functional group conversion.

## Introduction

Natural products (NPs) continue to inspire synthetic chemists to develop routes toward a variety of interesting molecules with important, clinically useful functions.^1^ Comprehensive genome sequence analysis has revealed that the encoded genes responsible for NP biosynthesis reside in biosynthetic gene clusters (BGCs).^2,3^ These BGCs harbor novel and unusual biocatalysts that could potentially be applied in the synthesis of a range of targets.^4,5^ If the substrate range of the native biocatalyst is too narrow for a desired function, engineering techniques such as directed evolution can be employed to expand its synthetic utility.^6,7^

**Figure 1:**
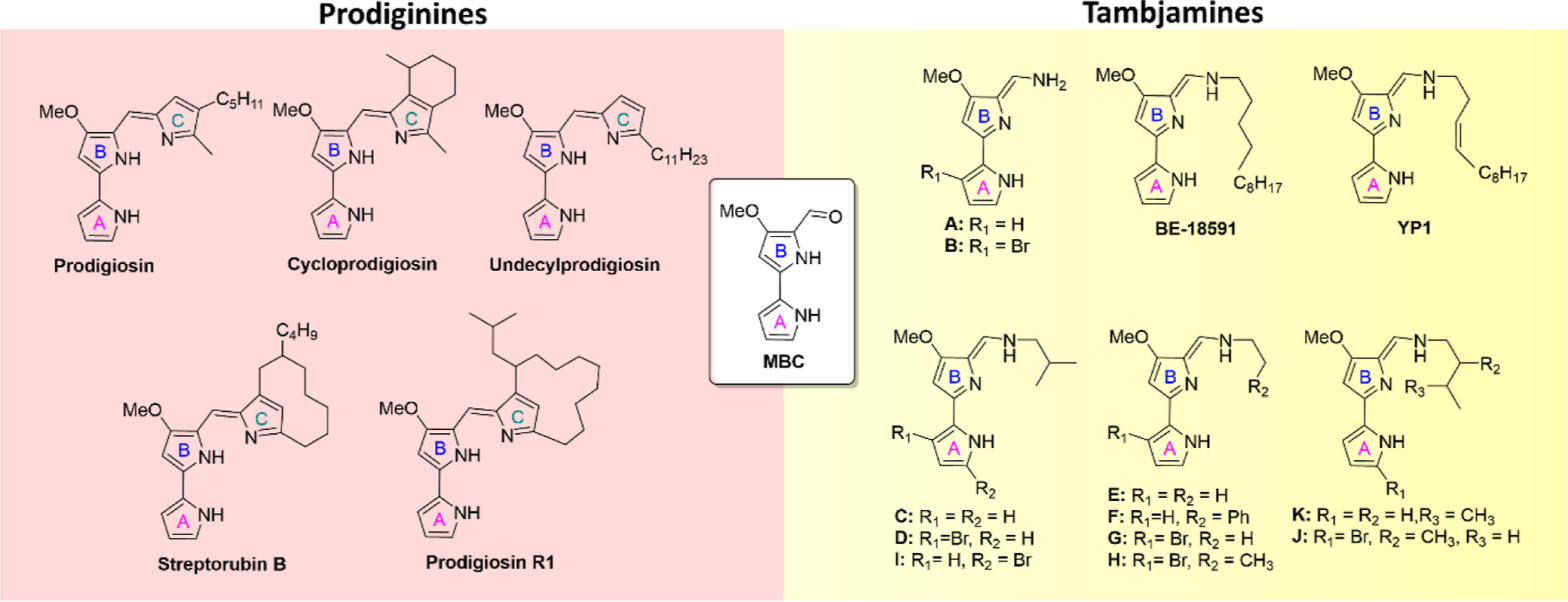
Structures of prodiginine and tambjamine NPs produced by the biosynthetic pathways of a number of organisms, highlighting the conserved MBC core.

Prodiginines are a class of secondary metabolites found in various organisms including the prodigiosin-producing *Serratia* sp. (*pig* cluster) and *Hahella chejuensis* (*hap* cluster), as well as *Streptomyces coelicolor* (*red* cluster), which predominantly produces undecylprodiginine, and *Streptomyces griseoviridis* (*rph* cluster), which produces prodigiosin R1 (Figure 1).^8−11^ They are structurally related to tambjamines (A-K, BE-18591, YP1) through a 4-methoxy-2,2′-bipyrrole-5-carbaldehyde (MBC) core, which is conserved throughout the entire prodiginine and tambjamine NP families (Figure 1). Extensive analysis identified a conserved set of homologous genes responsible for MBC biosynthesis within their respective BGCs. To form prodigiosin, the MBC core is condensed with another intermediate, 2-methyl-3-*n*-amyl-pyrrole (MAP); the first reported extraction of this NP was from the bacterium *Serratia marcescens*. Alternatively, a MAP derivative can be used.^12^ However, in the case of tambjamines, the MBC intermediate is condensed with an amine to form an enamine moiety in place of the third pyrrole ring. The biosynthetic production of these secondary intermediates can differ, along with the enzymes utilized for their production.

Along with the prodiginine and tambjamine families, NPs’ with pyrroles built into their scaffolds are ubiquitous in nature. The planar, electron-rich ring is able to form hydrogen bonds, chelate metal ions, and participate in π-stacking interactions.^13^ These five-membered *N*-heterocyclic-containing products often exhibit antimicrobial, antiviral or anticancer bioactivity, which has led to their use as therapeutic agents.^14−16^

A yellow-pigmented alkaloid, identified as tambjamine YP1 (YP1), was extracted from *Pseudoalteromonas tunicata*.^17^ Bioinformatic analysis and sequence alignment with homologous red and pig proteins helped to identify a distinct BGC designated as the “*tam*” cluster, which is responsible for YP1 biosynthesis (Figure S1).^18^ Of the 19 genes predicted, only a few have been expressed and their encoded enzymes characterized. The biosynthesis of YP1 requires two converging pathways; the first pathway produces the MBC core, whereas the second pathway produces the fatty amine tail. Since MBC biosynthesis is highly conserved, the genes required to make this bipyrrole core can be confidently predicted and annotated. However, the production and attachment of the unsaturated fatty amine tail are less well-understood.

The initial aim of this study was to characterize the formation of the fatty amine precursor to YP1. It was originally proposed that this second pathway in YP1 biosynthesis was initiated by AfaA, a fatty acid CoA ligase (FACL) not found within the “*tam*” cluster.^19^ Our recent investigations showed that another enzyme, *Pt*TamA [a di-domain enzyme comprising a class I adenylation (ANL) domain fused to an acyl carrier protein (ACP)], found within the “*tam*” cluster catalyzed this step. This enzyme uses adenosine triphosphate (ATP) and a C_12_ fatty acid (FA) to catalyze the formation of a C_12_ adenylate, which is then captured by the 4′-phosphopantetheine (4′-PP)-modified ACP domain to form an acyl-ACP-bound *Pt*TamA.^20,21^ This *Pt*TamA analysis led us to question how the bound acyl chain is released for further downstream tailoring. Two other enzymes in the BGC (*Pt*TamH and *Pt*TamT) are predicted to be involved in the formation of the amine tail after *Pt*TamA, although their exact function is yet unknown. Here, comprehensive sequence, spectroscopic, and chemical analysis revealed that *Pt*TamH is the unusual fusion enzyme that carries out the acyl chain off-loading function. We also show that recombinant *Pt*TamA and *Pt*TamH act together to convert fatty acids to the corresponding amines.

## Results and Discussion

### Sequence Analysis

An initial bioinformatic study on *Pt*TamH was performed to identify and characterize its catalytic domains. The deposited amino acid sequence of *Pt*TamH (941 aa, 104 kDa, NCBI reference sequence: WP_009837236.1, Uniprot: A4C5V8) is annotated as a di-domain enzyme, with an N-terminal, pyridoxal 5′-phosphate (PLP)-dependent transaminase (TA) type III domain and a C-terminal, amino acid dehydrogenase domain (Figure S2). Approximately 50 amino acids with no annotated function connect these two domains. An exhaustive phylogenetic analysis performed using the ConSurf^22−26^ server identified *Pt*TamH homologues from other *Pseudoalteromonas* species including *P. citrea* and *Pseudoalteromonas* sp.A25. More distantly related homologues with similar domain functions/organizations are present in *Chitinimonas*, *Paucibacter,* and *Streptomyces* species (Figure S3). It is currently unclear whether these distant homologues are involved in tambjamine biosynthesis or the closely related prodiginine biosynthetic pathway.^27^

While the N-terminal domain was confirmed to be a PLP-dependent ω-transaminase^28^ (ω-TA, residues M_1_-K_500_) by BLASTp, further sequence analysis suggests that the C-terminal domain (residues K_547_–S_941_) is more accurately described as a thioester reductase (TR). The proposed TR domain of *Pt*TamH shares sequence homology with acyl-ACP reductases (AARs) involved in alkane biosynthesis. Such homologues originate from cyanobacterial species including *Synechococcus elongates*(29) (28.3% identity), *Synechocystis* sp. PCC6803 (27.6% identity), and *Nostoc punctiforme*(30) (27.2% identity). Following multiple sequence alignments, the canonical nucleotide-binding motif GxxGxxG (G_707_xxG_710_xxG_713_) and a putative catalytic cysteine (C_887_) were identified in *Pt*TamH, as well as other *Pseudoalteromonas* homologues (Figure S4). As reported in the literature^31^ (and further evidenced by the crystal structure solved by Gao et al., PDB: 6JZY), the active residue C_294_ in *S. elongatus* AAR (*Se*AAR) is responsible for acyl chain transfer from a 4'-PP carrier such as *holo*-ACP or coenzyme A (CoA). The resulting thioester is optimally positioned for hydride attack by nicotinamide adenine dinucleotide phosphate hydrogen (NADPH), releasing an aldehyde. An identical mechanism was proposed by Warui et al. for *N. punctiforme* AAR.^30^ Given the apparent sequence similarity around this active site cysteine, it is plausible that the *Pt*TamH TR domain employs a comparable mechanism to liberate 4'-PP-bound intermediates as aldehyde products. Thus, from this sequence analysis, *Pt*TamH was putatively redefined a bifunctional ω-TA-TR fusion biocatalyst.

### Characterization of Recombinant *Pt*TamH

Recombinant *Pt*TamH was cloned into the pEHISTEV plasmid and expressed from *Escherichia coli* with a TEV cleavable HisTag (Figures S5–S7). The addition of 500 mM sorbitol to the growth media promotes correct protein folding, improving protein solubility.^32^ The soluble *Pt*TamH was isolated using a combination of cobalt-/nickel-immobilized metal affinity chromatography (IMAC) followed by size exclusion chromatography (SEC), which enabled the isolation of ∼1.5 mg/L. After initial isolation to characterize *Pt*TamH, and due to high protein purity after IMAC, SEC was omitted from the purification process, which led to a slightly higher protein yield of ∼3 mg/L. The SEC retention volume confirmed the homodimeric nature of *Pt*TamH (>200 kDa, see Figures S8 and S9); this observation was expected as PLP-dependent enzymes often form dimers or tetramers.^33^

### *Pt*TamH Is a Transaminase

**Figure 2:**
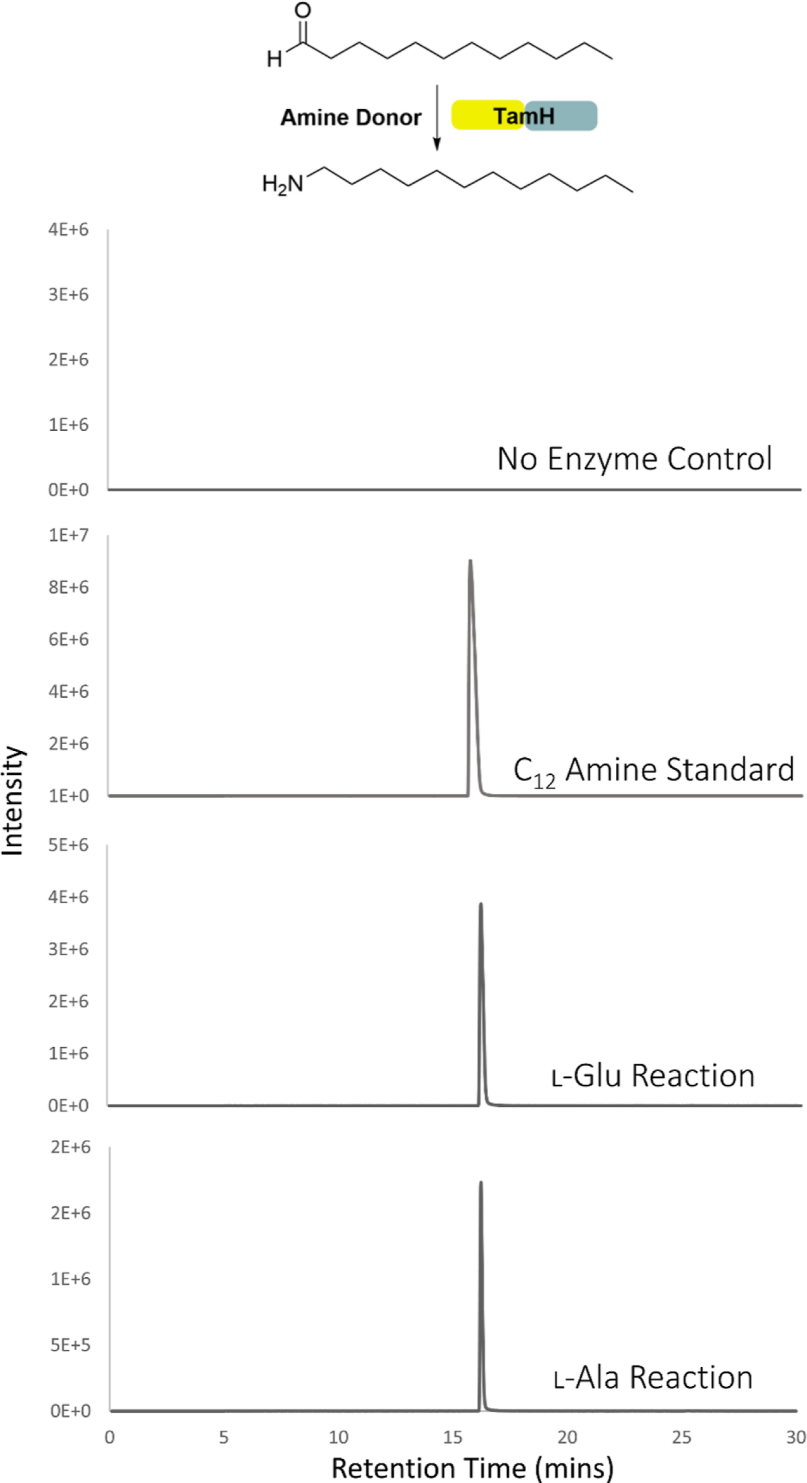
EICs for the C_12_ amine of the transamination reactions of *Pt*TamH (5 μM) in the presence of either l-Glu (5 mM) or l-Ala (5 mM) and C_12_ aldehyde (1 mM) for 24 h at 37 °C, leading to a peak with a retention time that corresponds to the amine standard. Each reaction was completed in triplicate.

The purified *Pt*TamH exhibited a strong yellow color and displayed a characteristic PLP spectrum when studied by UV–vis spectroscopy (Figure S10). As exemplified by the homologue CrmG, class III ω-TAs display a preference for either l-Glu or l-Ala as the amino donor; therefore, both amino acids were tested as substrates. The covalent binding of l-Glu to the key catalytic lysine (putative K_340_) was confirmed by UV–vis analysis, where the emergence of a peak at 330 nm indicates the formation of the pyridoxamine 5′-phosphate (PMP) intermediate (Figure S10). To confirm the activity of the predicted N-terminal ω-TA domain, *Pt*TamH was incubated with l-Glu or l-Ala and C_12_ aldehyde for 24 h, and the formation of the C_12_ amine product was monitored by liquid chromatography (LC) electrospray ionization-mass spectroscopy (ESI-MS). We observed a peak matching the retention time (16.2 min) of the C_12_ amine standard in both reactions. The extracted ion chromatograms (EICs) revealed an ion with *m*/*z* = 186.2222 Da, which matches with the predicted mass of C_12_ primary amine ([M + H]^+^, C_12_H_28_N) (Figure 2).

**Figure 3:**
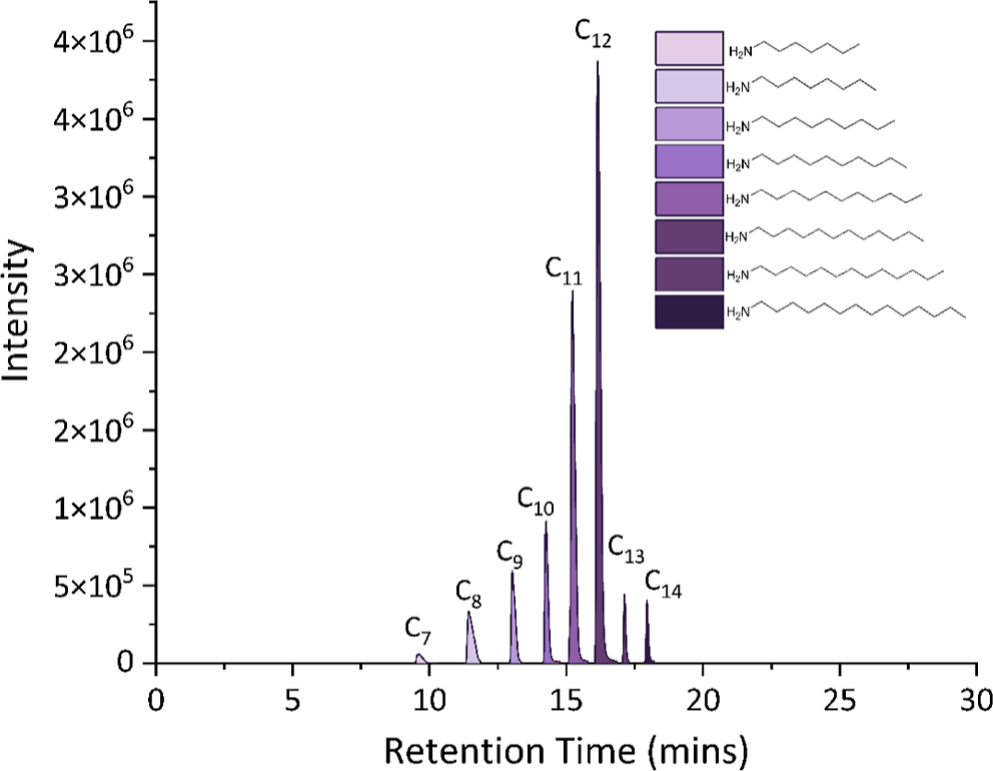
Monitoring of the EICs of C_7_–C_14_ amine products after incubation of the C_7_–C_14_ aldehyde with the *Pt*TamH TA domain in the presence of l-Glu. Each reaction was completed in triplicate.

Since the NP YP1 contains a C_12_ tail, it was hypothesized that *Pt*TamH prefers C_12_ aldehyde as its primary substrate. We therefore probed the chain-length specificity of the *Pt*TamH ω-TA domain using LC ESI-MS. When *Pt*TamH was screened against a palette of aliphatic fatty aldehydes (C_6_–C_14_), the corresponding amine products (except C_6_) were detected, and a clear preference for C_12_ aldehyde was also observed (Figures 3 and S11). These data show that *Pt*TamH displays a broad acyl chain selectivity between C_7_–C_14_ fatty aldehydes.

### *Pt*TamH TR Domain

After demonstrating the activity of the *Pt*TamH N-terminal ω-TA domain, we next focused on the predicted C-terminal TR domain. Inspired by the study of coelimycin biosynthesis by Awodi et al.—which involves an analogous TR-aminotransferase cooperativity^34^—*Pt*TamH was incubated with C_12_ CoA and either NADH or NADPH in the presence of l-Glu. Furthermore, the reaction mixture was supplemented with 200 mM KCl and 10 mM MgCl_2_ as both magnesium and potassium ions have been found to drastically improve the activity of homologous AARs.^29−31^ However, no C_12_ amine product was observed in either the NADH or NADPH reactions. This suggested that the *Pt*TamH TR domain is either inactive or does not accept acyl-CoA substrates (Figure S12).

### Coupled TamA-TamH Cascade

The inability of the *Pt*TamH TR domain to accept a free acyl-CoA thioester substrate was not entirely unexpected. As previously discussed, the tambjamine YP1 BGC encodes *Pt*TamA, a di-domain ACP-ANL natural fusion. Our earlier investigations showed that the *Pt*TamA ANL domain can activate carboxylic acids of various chain lengths (C_6_–C_14_) and attach them to the 4′-PP arm of the fused ACP domain.^20,21^ The presence of this enzyme suggests that *Pt*TamH may be acyl-ACP dependent, transferring the acyl chain directly from the *Pt*TamA acyl-ACP domain to the *Pt*TamH TR domain.

**Figure 4:**
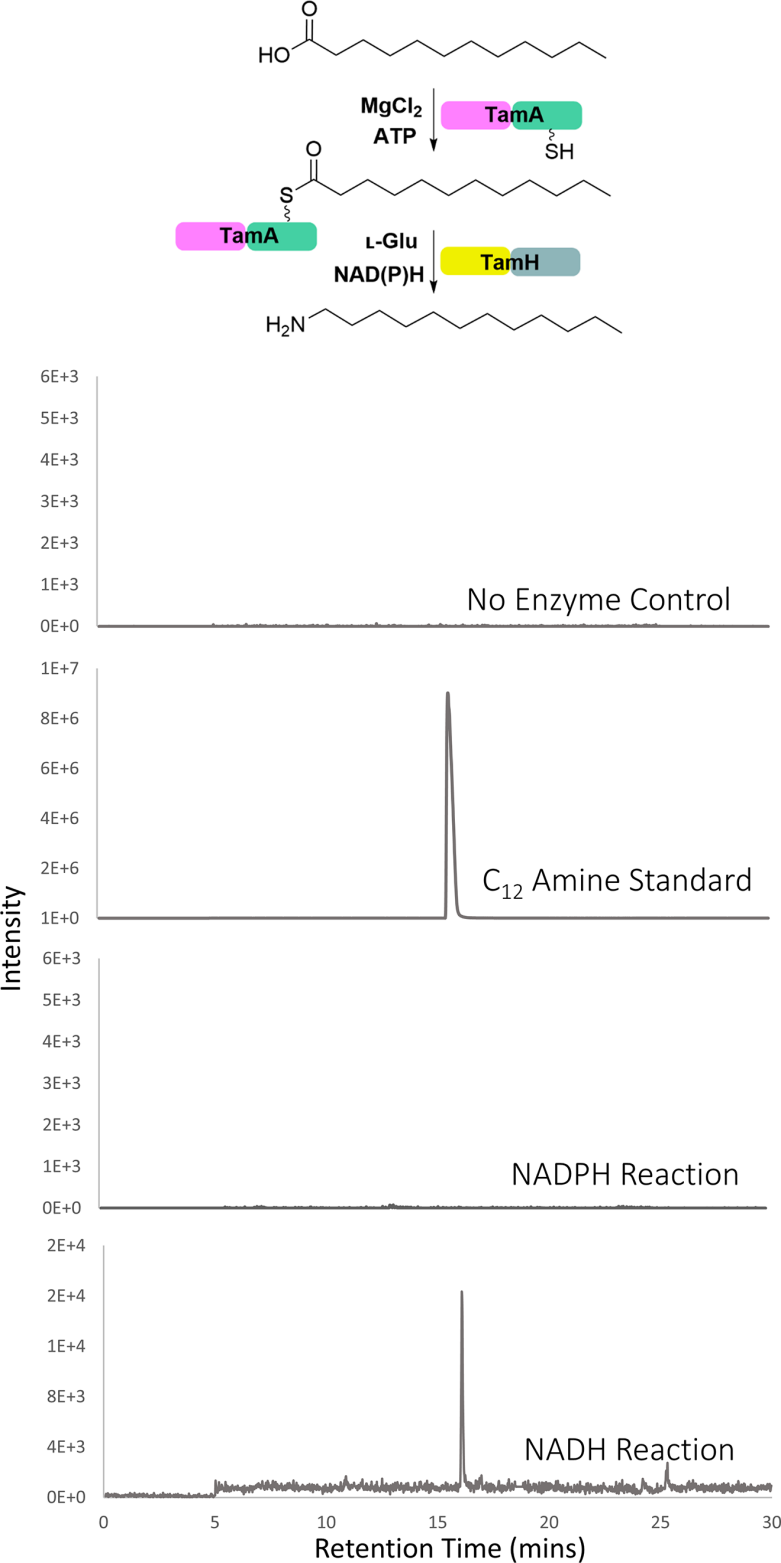
EICs for the coupled cascade of *Pt*TamA and *Pt*TamH, transforming the C_12_ acid to the C_12_ amine, through incubation with l-Glu, C_12_ acid, KCl, MgCl_2_, ATP, PLP, and NADH or NADPH for 24 h at 37 °C, leading to a peak with a retention time that corresponds to the amine standard in the NADH reaction. This reaction was completed in triplicate.

**Figure 5:**
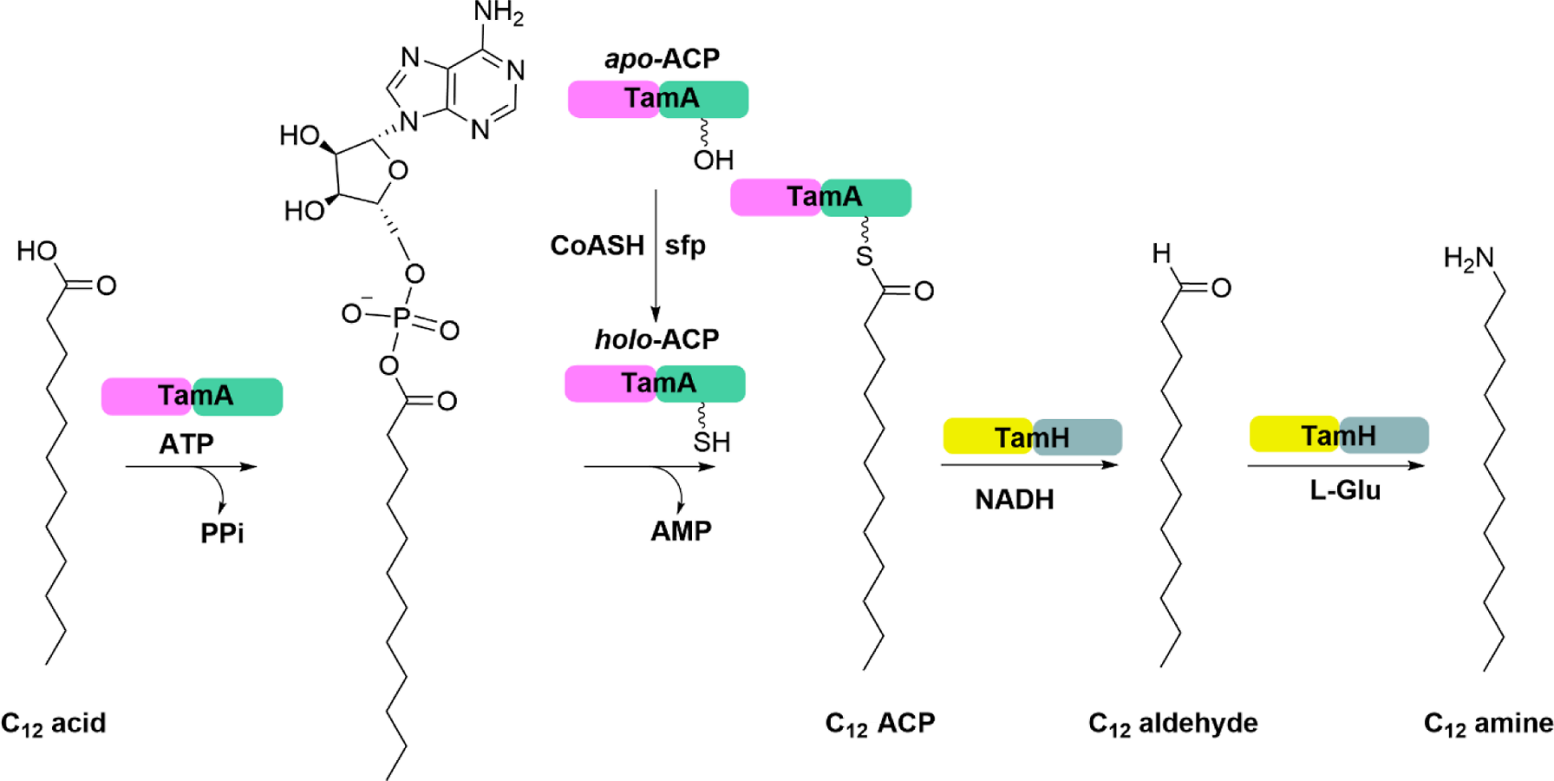
Coupled *Pt*TamA-*Pt*TamH cascade for the formation of the long-chain C_12_ amine product.

Therefore, a cascade reaction was performed using purified *Pt*TamA (Figure S13), prepared with the ACP domain in its 4'-PP-activated *holo*-form.^20,21^*Pt*TamA was incubated with *Pt*TamH in the presence of C_12_ acid, MgATP, KCl, NADH or NADPH, and l-Glu, and the production of the corresponding C_12_ amine was monitored by LC ESI-MS (Figure 4). A peak with *m*/*z* = 186.2222 Da was detected by LC ESI-MS in the NADH reaction. In contrast, no amine is produced in the NADPH reaction, illustrating that *Pt*TamH is NADH-specific (Figure 4). The amine product was also absent in the control reactions. Furthermore, the gas chromatography (GC)–MS study of the *Pt*TamA-*Pt*TamH cascade in the absence of l-Glu enabled the detection of the C_12_ aldehyde intermediate (Figure S14). Taken all together, our data not only confirm that the *Pt*TamH TR domain is catalytically active but also confirm that it exhibits an innate specificity for the C_12_ ACP substrate. The four domains are therefore working in concert to convert the acid to an amine. The C_12_ acid is activated by the *Pt*TamA ANL domain^35,36^ in an ATP-dependent reaction that leads to the formation of the C_12_ adenylate intermediate. The *Pt*TamA *holo*-ACP 4'-PP thiol acts as a nucleophile, attacking the C_12_ adenylate and releasing AMP. Once captured, the C_12_ ACP is then reduced by the *Pt*TamH TR domain using NADH, forming the C_12_ aldehyde. The final step involves transamination to give the final C_12_ amine, catalyzed by the *Pt*TamH TA domain (Figure 5). The experimental data supports the initial bioinformatic annotations of both *Pt*TamA and *Pt*TamH and show that the conversion of C_12_ FA to C_12_ fatty amine is catalyzed by this cascade of di-domain fusion biocatalysts (Figure 5).

### Predictive Structural Modeling

**Figure 6:**
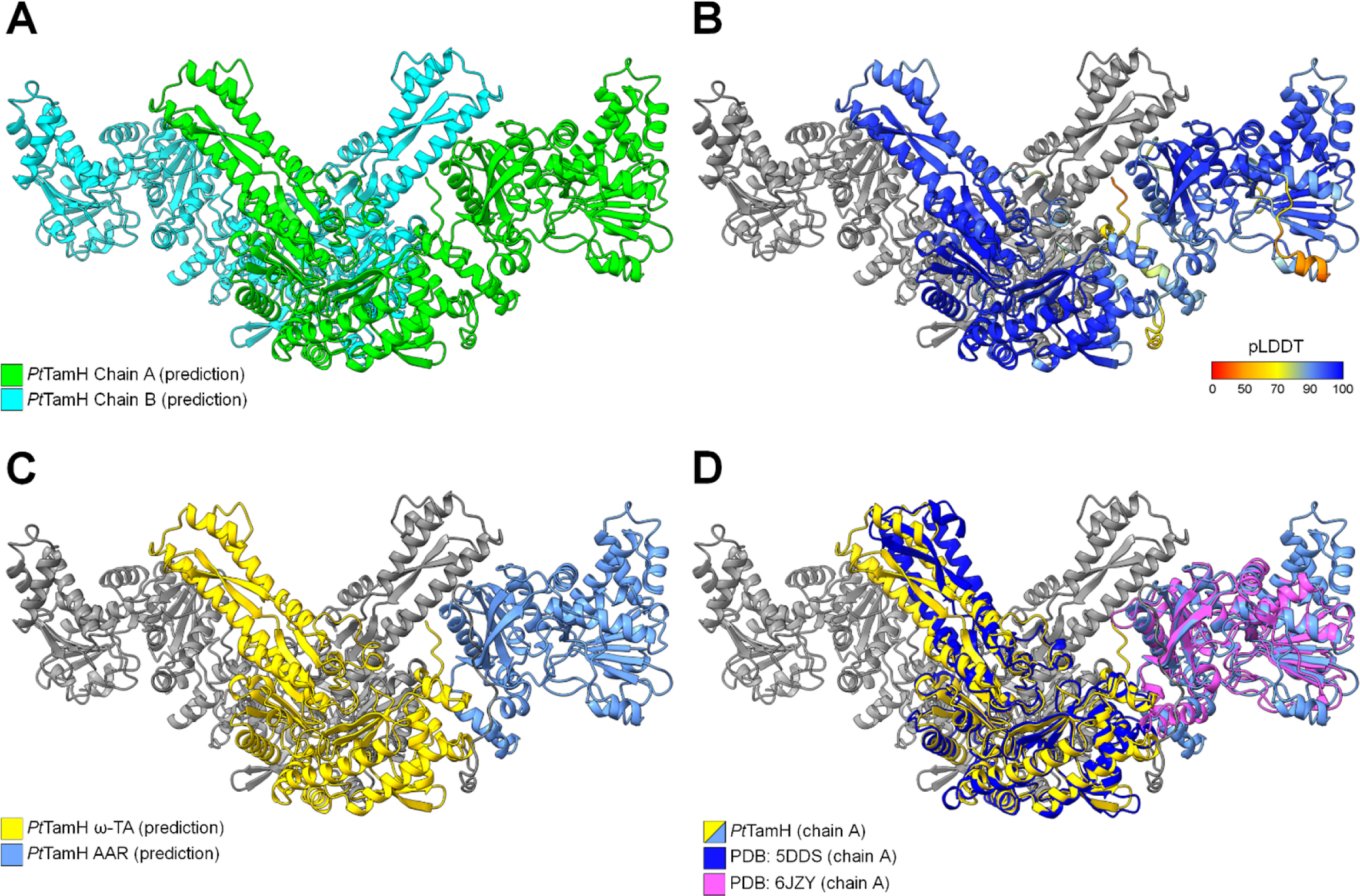
Predicted homodimeric model of PtTamH. (A) *Pt*TamH monomer chains A and B highlighted in green and cyan, respectively. (B) pLDDT score of the *Pt*TamH monomer. (C) *Pt*TamH ω-TA and TR domains highlighted in yellow and steel blue, respectively. (D) Closest known structural homologues CrmG (PDB: 5DDS, RMSD: 0.994 Å between 323 pruned atom pairs) and *Se*AAR (PDB: 6JZY, RMSD: 1.13 Å between 209 pruned atom pairs) superimposed on the predicted *Pt*TamH monomer chain A.

Following experimental characterization, we sought to understand the structural logic underpinning the activity of *Pt*TamH. To this end, a head-to-head homodimeric model of *Pt*TamH was predicted using the accurate deep-learning program ColabFold^37−39^ (Figure 6, see also the Supporting Information for further details) and studied by molecular dynamics simulation (MDS). The predicted ω-TA domain shares structural homology with several ω-TA crystal structures including YgjG^40^ (PDB: 4UOX, 30.1% identity), PigE^41^ (PDB: 4PPM, 30.5% identity), ArgD (PDB: 1VEF, 31.4% identity), and CrmG^42^ (PDB: 5DDS, 29.0% identity), with the latter displaying the highest sequence coverage of the homologues identified (90%, Figure 6D). Evolutionary conservation analysis and superimposition of the aforementioned ω-TA structures show that the PLP-binding core is highly conserved, including the key PLP-binding residue K_340_ (Figure S15A,B). This critical lysine covalently binds PLP via a Schiff base linkage, thereby enabling the coenzyme to participate in transamination (Figure S15B).^43,44^ Furthermore, the ω-TA domains are predicted to comprise the homodimeric interface; identical interfaces exist across all solved ω-TA structures to date, and ω-TA dimerization is essential for defining the active site. Using this model, the C_12_ external aldimine could be docked in the predicted ω-TA binding tunnel, with the PMP moiety flanked by K_340_ and F_206_ and the alkyl chain extending toward a hydrophobic pocket comprising F_51_, L_470_, and L_473_ from chain A and M_365_ from chain B (Figure S15B,C).

The predicted *Pt*TamH TR model and the crystal structure of *Se*AAR could be comfortably superimposed (Figure 6D), enabling further structural study. *Pt*TamH TR is modeled with a classic Rossmann fold for nucleotide binding. An alkyl-binding pocket was also inferred by structural homology and topological analysis (Figure S16A), encompassing several hydrophobic residues (including F_550_, L_554_, I_555_, L_560_, I_563_, L_591_, and L_874_) complementary to the lipophilicity of *Pt*TamH’s natural substrate (Figure S16B). Like *Se*AAR, the putative catalytic cysteine C_887_ is positioned at the intersection between the NADH- and alkyl-binding subdomains. Evolutionary conservation analysis suggests that the active core of the reductase is highly conserved, including residues involved in nucleotide binding as well as C_887_ itself (Figure S17A). By comparison, the residues predicted to stabilize the acyl chain display greater variability, which may suggest an additional substrate-recognition role for this pocket. With this knowledge, both NAD^+^ and C_12_ aldehyde ligands were docked in this predicted active site. The top-ranked outputs position the aldehyde carbonyl near C_887_ and the NAD^+^ pyridine (Figure S17B), with the alkyl chain extending into the predicted hydrophobic pocket (Figure S17C,D). These poses are remarkably similar to the ligand orientation in the *Se*AAR crystal structure (Figure S17E) and suggest a conserved active site architecture within this class of reductases.

The fully predicted *Pt*TamH homodimer maintained its structural integrity over the course of a 10 ns MDS (see Figures S18 and S19 in the Supporting Information for detailed analysis). The simulated 208 kDa complex positions the TR domains laterally from the ω-TA core. Studying the electrostatic properties of *Pt*TamH revealed an electropositive surface on the solvent-exposed substrate channel of the TR domain, which may underpin the recognition/docking of the comparatively acidic *Pt*TamA ACP (Figure S20).^45^ Thus, the predicted domain organization of *Pt*TamH would permit the facile transfer of acyl intermediates between the ACP of *Pt*TamA and the TR domain of *Pt*TamH. Taken alongside the experimental data, the *Pt*TamH TR domain can be confidently classified as an AAR. The fascinating predicted architecture of *Pt*TamH makes it an attractive target for further structural studies.

## Conclusions

The application of biocatalysts for the synthesis of a range of high-value molecules (e.g., sitagliptin and islatravir)^46^ is gaining in popularity.^47−49^ Once an enzyme has been discovered, its substrate specificity can be engineered to widen the substrate scope for a bespoke synthetic application.^6^ A rich source of these enzymes has been NP biosynthetic pathways which have evolved to efficiently transform simple building blocks into complex structures with a myriad of functionalities.^50^ These NPs are often encoded in BGCs where each enzyme plays a specific role in the stepwise transformation along a linear path. Along with these discrete, single-domain biocatalysts, the polyketide and nonribosomal peptide classes of NPs are members of a large family whose biosynthesis is driven by large, multidomain assemblies.^45,51,52^ The fused domains within these molecular machines have clearly evolved to efficiently transfer intermediates between active sites. A key player within the complex is the acyl-ACP substrate which relays covalently tethered substrates between domains.

The tambjamine YP1 BGC encodes a pathway to convert long-chain FAs to the essential hydrophobic fatty amine tails of these biologically active NPs. The details of this part of the pathway were unclear until we reported that the *Pt*TamA enzyme is a di-domain fusion of an N-terminal ANL to a C-terminal ACP.^20,21^ However, the details involved in the downstream processing of the novel *Pt*TamA acyl-ACP intermediate were unknown. In this study, the putative biocatalyst *Pt*TamH was analyzed using comprehensive bioinformatic analysis. This predicted *Pt*TamH to also be a di-domain fusion with an N-terminal domain that displays high-sequence homology to a class III PLP-dependent TA. This is fused to a C-terminal domain whose sequence and structural homology suggest that it is a member of the AAR family. To assign a function to the individual domains, the recombinant *Pt*TamH was initially shown to bind PLP and catalyze the conversion of C_12_ aldehyde to the corresponding C_12_ amine. The preferred amine donor was found to be l-Glu (but it can also accept l-Ala to a lesser extent), and it also displays a broad substrate promiscuity by being able to accept C_7_–C_14_ aldehydes. In future, the synthetic utility of this TA could be further expanded by studying its activity with smart amine donors such as cadaverine, *o*-xylylenediamine, and *N*-phenylputrescine (NPP).^53−55^

The outstanding activity to be defined was that of the origin of the aldehyde substrate, and we predicted that the TR domain would catalyze the reduction of an acyl-thioester substrate, but *Pt*TamH was unable to convert C_12_ CoA to the corresponding C_12_ aldehyde. However, we established that the *Pt*TamH TR domain was catalytically active by successfully constructing a biocatalytic cascade which converted the C_12_ acid to the corresponding amine. Moreover, we also detected the formation of an aldehyde intermediate by omission of the amine donor. This suggests the four domains, present as two fusions within *Pt*TamA and *Pt*TamH, worked together and that the *Pt*TamH TR domain requires a specific acyl-ACP-bound thioester substrate. Defining the function of *Pt*TamH, and coupling it with *Pt*TamA, finally resolves the origin of the fatty amine tail of tambjamine YP1.^18,20,21,56^

Further work on the *Pt*TamA-*Pt*TamH system could be carried out to fully explore the substrate scope of this novel biocatalytic cascade. This would be enabled by determination of the three-dimensional structures of both enzymes; our sequence and structural analyses has provided initial insights into the chemistry and logic underpinning *Pt*TamH-*Pt*TamA cooperativity. The dimeric *Pt*TamH is >200 kDa and is also an excellent candidate for cryogenic electron microscopy studies.^57^ Further issues to be resolved include the molecular details of how each domain interacts with each other and how substrates and products navigate between catalytic sites. Tambjamine YP1 contains an oxidized acyl chain (with *cis* geometry between C_3_ and C_4_), and *Pt*TamT within the *P. tunicata* BGC has been proposed to perform this oxidation. The active biocatalytic cascade described here will allow functional analysis of this remaining useful enzyme. Furthermore, along with YP1, Picott et al. have recently characterized the macrocyclic tambjamine derivative MYP1 produced by *P. citrea*. Initial analysis identifies *Pt*TamA and *Pt*TamH homologues [TreaA (Uniprot: U1J4V2) and TreaH (Uniprot: U1KHB2)], in the BGC of this organism, which both show 71% identity to *Pt*TamA and *Pt*TamH, respectively. Our work lays the foundation to reveal the similarities and differences between the two biosynthetic pathways.^56^

Members of the ANL family are versatile biocatalysts that can be used to activate a range of FA substrates and prepare useful amides, esters, and thioesters.^58^ New tools such as the database RetroBioCat should become a useful resource to incorporate such adaptable biocatalysts into synthetic routes for the preparation of a range of target molecules.^59^ The work described here suggests that the functional group conversion displayed by the *Pt*TamA–*Pt*TamH biocatalytic cascade would be a valuable addition to this biocatalyst repository.

## Supporting Information Available

Details of the YP1 biosynthetic pathways; sequence alignments; gene cloning; enzyme purification; enzyme assay; mass spectrometry analysis; and predictive modeling, docking, and simulation (PDF)

## Abbreviations

4′-PP: 4′-phosphopantetheine
AAR: acyl-ACP reductase
ACP: acyl-carrier protein
ANL: adenylation
ATP: adenosine triphosphate
BGC: biosynthetic gene cluster
EIC: extracted ion chromatogram
FA: fatty acid
FACL: fatty acid CoA ligase
IMAC: immobilized metal affinity chromatography
MAP: 2-methyl-3-*n*-amyl-pyrrole
MBC: 4-methoxy-2,2′-bipyrrole-5-carbaldehyde
NP: natural product
NRPS: nonribosomal peptide synthase
PLP: pyridoxal 5′-phosphate
PKS: polyketide synthases
TA: transaminase
TR: thioester reductase
SEC: size exclusion chromatography
