# Supplementary material for: Coupled Natural Fusion Enzymes in a Novel Biocatalytic Cascade Convert Fatty Acids to Amines
Source: ACS Catal. 2022 Oct 5;12(20):12701–10. doi: 10.1021/acscatal.2c02954 (PMC9594044; doi:10.1021/acscatal.2c02954)
Supplement: Supplementary file 1 — cs2c02954_si_001.pdf [file cs2c02954_si_001.pdf]

Supplementary Information

Coupled natural fusion enzymes in a novel biocatalytic cascade convert fatty acids to amines

Shona M. Richardson#, Piera M. Marchetti#, Michael A. Herrera#, and Dominic J. Campopiano*

AUTHOR ADDRESS: School of Chemistry, David Brewster Road, The University of Edinburgh, Edinburgh, EH9 3FJ, United Kingdom

# These authors contributed equally.

* Corresponding author

Table of Contents

Materials and Methods: 1

Additional Notes on Predictive Modelling, Docking and Simulation: 4

Supplementary Figures: 5

Figure S1: Tambjamine Biosynthetic pathway. 5

Figure S2: *Pt*TamH didomain sequence. 6

Figure S3: Percent identity matrix of the closest identified *Pt*TamH homologues. 7

Figure S4: Multiple sequence alignment of *Pt*TamH TR (and known *Pseudoalteromonas* homologues) with cyanobacterial acyl-ACP reductases (AARs). 8

Figure S5: Sequence of *Pt*TamH gene. 9

Figure S6: *Pt*TamH recombinant protein sequence. 10

Figure S7: *Pt*TamH pEHISTEV plasmid map. 11

Figure S8: *Pt*TamH purification. 12

Figure S9: Superdex HiLoad 16/60 S200 calibration curve. 13

Figure S10: UV-Vis PLP binding spectrum of *Pt*TamH. 14

Figure S11: Extracted ion chromatograms (EICs) of C_7-C_{14} amine products after incubation with *Pt*TamH. 15

Figure S12: Attempted *Pt*TamH + C_{12}-CoA reaction for the formation of the C_{12} amine product. 16

Figure S13: *Pt*TamA purification. 17
Figure S14: Detection of C_{12} aldehyde from a *Pt*TamH + *Pt*TamA cascade lacking L-Glu. 18
Figure S15: *Pt*TamH ω-TA conservation analysis and ligand docking. 19
Figure S16: *Pt*TamH TR topological analysis. 20
Figure S17: *Pt*TamH TR conservation analysis and ligand docking. 21
Figure S18: Structural morphology of the *Pt*TamH complex during MDS. 22
Figure S19: A summary of the *Pt*TamH MDS. 23
Figure S20: APBS electrostatics analysis of *Pt*TamH and the *Pt*TamA ACP domain. 24
References: 25

Materials and Methods:

*Pt*TamH Cloning and Expression Tests

*Pt*TamH was cloned from *Pseudoalteromonas tunicata D2* genomic DNA into pEHISTEV[1] using restriction digest and ligation cloning with the following primers:

- *Pt*TamH Forward (NcoI) – ATTA**CCATGG**AAATTCGTGTTGGACAAGAAGTTTTAAGCCGAG
- *Pt*TamH Reverse (XhoI) – ATAT**CTCGAG**TCAGCTAGCGGCTTGCAAGGCGTCATATT.

Expression of this *Pt*TamH construct with a TEV-cleavable N-terminal 6 x His tag led to a large insoluble band, with very little soluble protein produced. The addition of sorbitol to the growth media *as per* literature[2] greatly increased protein solubility.

*Pt*TamH Expression and Purification

Chemically-competent BL21 (DE3) cells were transformed using the pEHISTEV *Pt*TamH expressing construct; successful transformants were screened on LB-agar plates supplemented with kanamycin (50 μg mL^{-1}). An overnight culture was subsequently prepared in kanamycin-LB media and incubated at 37 °C with agitation. A sample of the overnight culture was back-diluted to OD_{600} = 0.1 in kanamycin-LB media supplemented with 500 mM sorbitol in 100 mM potassium phosphate, pH 7.5. At OD_{600} = 0.6-0.8, protein expression was induced using 0.5 mM IPTG. The induced culture was incubated at 16 °C overnight with agitation. The biomass was harvested by centrifugation at 3500 xg for 15 minutes and stored at -20 °C until needed. The subsequent purification steps were carried out at 4 °C. The biomass was resuspended in lysis buffer (50 mM HEPES pH 8, 250 mM NaCl, 10 mM imidazole, 10 % glycerol, 25 μM PLP) and lysed by sonication for a total of 10 minutes (30 second pulse, 30 second cooldown). Cell debris was removed by centrifugation at 10,000 xg for 45 mins and the supernatant was clarified through a 0.45 micron filter. The cell-free extract was pumped through a 1 mL HiTrap TALON Crude column (Cytiva) using a 1 mL min^{-1} flow rate. The imidazole concentration was steadily increased remove any impurities before the protein was eluted with 250 mM imidazole. The fractions containing *Pt*TamH were combined and dialysed with 1 mg polyhistidine-tagged TEV protease against the dialysis buffer (50 mM HEPES, 250 mM NaCl and 10 % glycerol) for 2 hrs. The dialysed protein mixture was pumped through a 1 mL HisTrap HP (Cytiva) to remove the TEV protease and any uncleaved *Pt*TamH, and the untagged *Pt*TamH was recovered in the flowthrough. Fractions containing untagged *Pt*TamH were combined and the volume was

reduced using a 30 kDa MWCO centrifugal concentrator. ~3.0 mg mL^{-1} of high-purity *Pt*TamH could be routinely prepared using this IMAC strategy alone. For further characterisation, *Pt*TamH was injected onto a 120 mL Superdex S200 (Cytiva) size exclusion column (SEC) pre-equilibrated with buffer (50 mM HEPES, 250 mM NaCl and 10 % glycerol). All purifications were monitored by SDS-PAGE.

_Pt_TamH Aldehyde Reactions

Reactions contained 5 µM *Pt*TamH, 250 µM PLP, 5 mM amine donor (ʟ -Glu or ʟ -Ala) and 1 mM C_6-C_{14} aldehyde (from a 10 mM stock in DMSO) in Buffer (50 mM HEPES, 100 mM NaCl and 1 mM DTT). The reactions were incubated at 37 °C for 24 hrs in triplicate and alongside control reactions prior to LC ESI-MS analysis.

_Pt_TamH acyl-CoA Reactions

Reactions contained 5 µM *Pt*TamH, 250 µM PLP, 5 mM ʟ-Glu, 2 mM NADH/NADPH and 1 mM C_{12}-CoA in Buffer (50 mM HEPES, 200 mM KCl, 10 mM $MgCl_2$ and 1 mM DTT). The reactions were incubated at 37 °C for 24 hrs in triplicate alongside control reactions prior to LC ESI-MS analysis.

_Pt_TamA-_Pt_TamH coupled Reaction

Reactions contained 5 µM *Pt*TamH, 5 µM *holo-Pt*TamA, 250 µM PLP, 2 mM NADH/NADPH, 5 mM ATP and 1 mM C_{12} acid (from a 25 mM stock in DMSO) in Buffer (50 mM HEPES, 200 mM KCl, 10 mM $MgCl_2$and 1 mM DTT). The reactions were incubated at 37 °C for ~4 hrs before the addition of 5 mM ʟ-Glu. The reactions were then further incubated for ~20 hrs at 37 °C in triplicate and alongside control reactions before being analysed by LC ESI-MS.

Analysis of amine formation by LC ESI-MS

Amine reactions were quenched with a 1:1 volume of acetonitrile with 0.01% TFA and centrifuged at 17000 xg. 5 µL of supernatant was subjected to LC ESI-MS on a Synapt G2-Si Q-TOF (Waters) instrument with Phenomenex Jupiter C18 5 µm 300 Å LC column coupled to an ESI source. The LC gradient ran from 5% acetonitrile and 95% water with 0.1% formic acid to 95% acetonitrile over 30 min. The MS source was set at 120 °C, backing pressure 2 mbar and sampling cone voltage of 54 V. Extracted ion chromatograms (EICs) and masses were determined on MassLynx V4.1 software.

Detection of C_{12} aldehyde by GC-MS

Reactions contained 5 µM *Pt*TamH, 5 µM *holo-Pt*TamA, 250 µM PLP, 2 mM NADH, 5 mM ATP and 1 mM C_{12} acid (from a 25 mM stock in DMSO) in Buffer (50 mM HEPES, 200 mM KCl, 10 mM $MgCl_2$and 1 mM DTT) in the absence of ʟ-Glu amine donor. The reactions were incubated at 37 °C for 24 hrs in triplicate. The aldehyde product was extracted from the reaction mixture using an equivalent volume of EtOAc. The organic phase was sampled for GC-MS analysis using a Shimadzu QP2010 SE fitted with a Zebron ZB-FFAP capillary column (25 mm internal diameter [ID], 25 µm film thickness, 30 m length). 1 µL sample was injected (split 10:1 or 50:1, 230 °C inlet temperature using a Restek Topaz 3.5 mm ID quartz wool inlet liner) and chromatographically resolved under 1 mL min^{-1} constant helium flow using the following oven profile: 55 °C initial temperature (hold 2 minutes), 200 °C (20 °C min^{-1} ramp, hold 4 minutes), 240 °C (20 °C min^{-1} ramp, no hold). The MS was configured to detect ions over a range of 40-620 *m/z* following a 3.5 minute solvent delay. The ion source and transfer line temperatures were set to 180 °C.

Structure Prediction and Ligand Docking

All structural predictions were performed using ColabFold via AlphaFold2_advanced.ipynb. In brief, a deep multiple sequence alignment (MSA) was generated using MMSeqs2 prior to structure prediction using AlphaFold 2 (structural templates were not utilised for prediction). When appropriate, ColabFold was configured to perform homodimeric prediction. The output of the AlphaFold 2 structure module was recycled up to 6 times for refinement. For each

sequence, a total of 5 models were generated and ranked by Predicted Template Model score (pTM); Predicted Local Distance Difference Test (pLDDT) scores were also computed for each model to evaluate fold-level confidence. The best model was subsequently relaxed to eliminate steric clashes. Visual inspection was performed in UCSF ChimeraX (v1.3)[3] and PyMOL (v2.4), and electrostatic potentials were computed using APBS Electrostatics.[4] Topological analysis was performed using the CASTp 3.0 server.[5] For docking studies, both the ligand and receptor were prepared using AutoDockTools[6], and ligand docking was performed using AutoDock Vina (v1.1.2)[7, 8]. Ligand-receptor hydropathy surfaces were computed in BIOVIA Discovery Studio 2020.

Evolutionary Conservation Analysis

Evolutionary conservation analysis was performed using the ConSurf[13, 14, 15] server configured to build MSAs using MAFFT. 106 homologous sequences with identities ranging from 30-95% were compiled from UNIREF90 using the HMMER search algorithm. Conservation scores were calculated via the Bayesian method and visualised using UCSF ChimeraX 1.3.

Molecular Dynamics Simulation

Simulations were performed using GROMACS 2021.4.[9] Protein charges were computed using CHARMM36 all-atom forcefield.[10] The model was solvated in TIP3P water in a cubic box, and the net protein charge was counterbalanced using simulated sodium ions. The system was energy-minimised by sequential steepest descent/conjugate gradient descent and equilibrated to 300K and 1 bar using V-Rescale thermostat/Berendsen barostat. Following a 10 ns (5×10^6 time steps) production MD, the trajectory was re-centered with additional rotational and translational fitting. Further analysis was performed in GROMACS using gmx gyrate, gmx hbond and gmx rms. UCSF Chimera 1.16[11] was used for trajectory visualisation and for computing pairwise RMSDs.

Additional Notes on Predictive Modelling, Docking and Simulation:

Due to computational limitations, the *Pt*TamH was modelled in three parts using the ColabFold parameters outlined in the Materials and Methods. First, the *Pt*TamH ω-TA domain was accurately predicted as a homodimer (pLDDT: 90.27, pTM: 0.89); no plausible homomeric interface could be identified when docking the TR domain against itself (pTM < 0.6). Second, a separate prediction of the full *Pt*TamH monomer was generated to gauge the relative orientation of the ω-TA and TR domains; the top-ranked output was predicted with high confidence (pLDDT: 92.27, pTM: 0.87, see Fig. 6B in main text). The final, complete homodimeric model was created by superimposition of two *Pt*TamH monomers onto the predicted ω-TA dimer (RMSD: 0.395 Å between 510 pruned atom pairs). The structure was subsequently relaxed via a two-step steepest descent/conjugate gradient descent. Using AutoDock Vina and Vina forcefield, the C_{12}-external aldimine was docked in the active site of the ω-TA domain with a calculated binding affinity of -7.6 kcal mol^{-1}. Similarly, both the C_{12} aldehyde and NAD+ were docked in the TR domain with binding affinities of -3.5 kcal mol^{-1} and -10.1 kcal mol^{-1} respectively.

The complex was studied in a 10 ns (5 x 10^6 time steps) molecular dynamics simulation (MDS, see Methods and Materials). The interfacial contacts of the *Pt*TamH ω-TA domains are maintained over the course of the MDS (see figure S18). In particular, the homomeric complex is stabilised by an average of 26 ± 5 hydrogen bonds, the majority of which (58%) occur within a distance of 2.67-2.93 Å. (figure S19A-B). While the average radius of gyration (R_g = 4.88 ± 0.03 nm, R_g max-min = 0.197 nm) suggests that the *Pt*TamH complex is very stable, pairwise RMSD analysis reveals that the fusion enzyme may exhibit a moderate amount of conformational flexibility, with RMSDs as high as 6 Å occasionally observed (figure S19C-D). Throughout the MD trajectory, the TR domains remain oriented laterally from the ω-TA dimer interface with the putative substrate channel readily accessible for ACP docking. An accurate model of the *Pt*TamA ACP was also predicted (pLDDT: 87.98), and complementary electrostatic surfaces between the ACP and *Pt*TamH TR were identified using APBS electrostatics (see S20A-B).

Supplementary Figures:

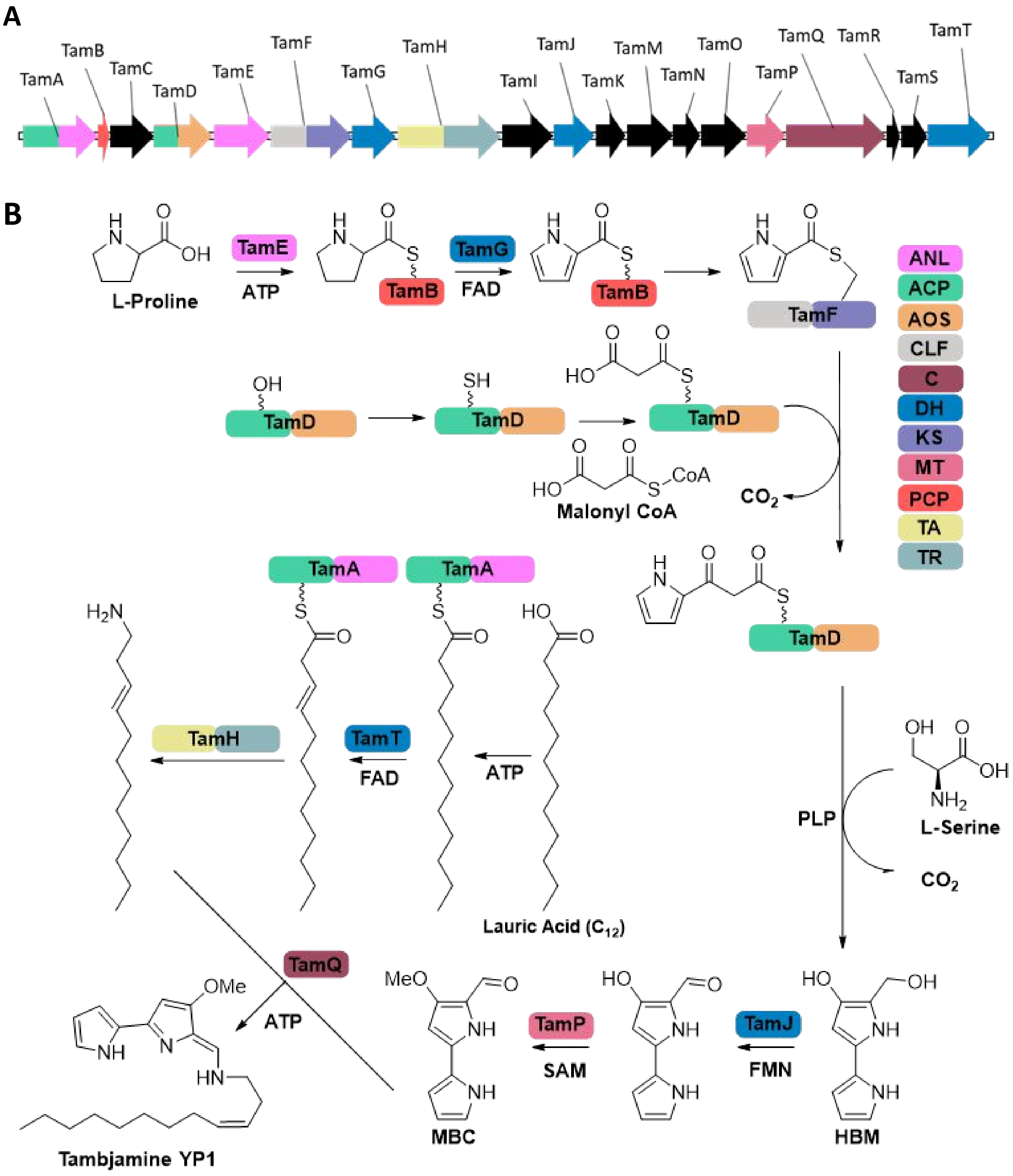

Figure S1: Tambjamine Biosynthetic pathway.
A. Biosynthetic gene cluster (BGC) designated the '*tam*' cluster responsible for the production of tambjamine YP1 in the *Pseudoalteromonas tunicata* organism. **B.** Predicted biosynthetic pathway for assembly of tambjamine YP1, shown as the production of the MBC core and free unsaturated amine which are then condensed together by TamQ to form the final molecule.

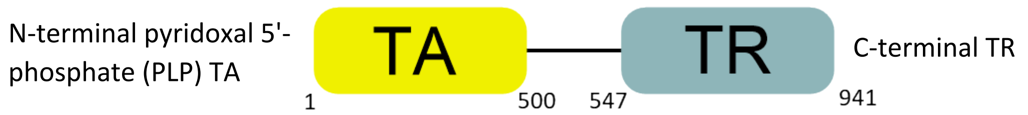

MQIRVGQEVL SRESLLESAG LLAQYIRAQG DMLTWQKEDE RIEVLDMVGG FGSTLLGHNH PELLATMQQS LSSLRPMWVQ 81
GAERPVAKQL RNALAQKLLR ETGKKYSIVL LNTGTEAVEA GLKHAQYEFF QRLQHIQQHC DTNWREFKLR LARNEIQLTS 161
EFYLECERLL QQEPIESLEE LQRAVQMRNQ AVFNSSGKIA AFKGDFHGKT RGSLATTYNR DARLPFIANN PDAIFIDDEA 241
QFKATLASWQ KAYFTIEFAP LRLQKKPVNL LTALIYEPIQ GEGGVRPLNA RYCALLNELK LSHPDVAIIA DEIQCGLGRT 321
GQFLESQAIN TPNDYITLAK SLGGGLCKIS ALAVEQTRYH EEFSMLHSTT FADDDLSSAV ALKTLAILER DELTLKAAHL 401
GEQFTTALNA LALEYPDMIA DIRGKGCMLG IELAAQENHP SATIAGLDEQ NMLAMAIAGH LLHRHHIRVL PSLGKRRVLR 481
LQPSAYLDAA NIALVVDALK **ETFELIRHHH VATLLAHLIH TDKPHSAFAT AYQHPIREEA IPAGVE**KVGF ISHLIDEESL 561
NEIDPSWRLF EGYEQEELNQ HILPITVPGV LARRLVTSAT GRKIELVLYG IQMDAESIEA DRRFNQAKIV RAQVNEAYRL 641
AREEGCRLVG FGGYTSIVTN NCCDYYYNEP ATTSGNALTV AASINTILNS AQDHGINLAK ATVAICGAAG NIGQVHSAIL 721
AKHCHKLLLI TRNVSANNMA MTLNMICEQL YQAVSQDQQG GILVSICRDM LASRIGHEAP KVLIDELKEA LLARQLVRIS 801
EHFNDCQQAD IIVSNTSSPT TVIDAQHVNA HKPVLISDVA VPRDVPVDIV NARPNIKLIR GGVVNLPINP SFTLPGMLLP 881
TGQVYACCGE TMLLGLAGAF SHFSMGALTC EQVEQVQALA AIHGFELNQE KPQYDALQAA S 941

941 Amino Acids, Mw = 104 kDa

Figure S2: *Pt*TamH didomain sequence.
Obtained from NCBI with the N-terminal transaminase (TA) domain shown in yellow, the C-terminal thioester (TR) domain shown in blue and the didomain linker shown in black.

	1	2	3	4	5	6	7	8
1 **A0A243SAX0**, *Streptomyces swartbergensis*	100.0	85.7	85.3	31.1	30.8	31.8	36.7	33.9
2 **A0A3Q9WFQ1**,*Streptomyces nigrescens*	85.7	100.0	85.5	31.1	30.5	31.3	36.4	34.0
3 **A0A059W5R7**, *Streptomyces albulus*	85.3	85.5	100.0	30.7	30.2	31.0	36.1	34.7
4 TamH, *Pseudoalteromonas tunicata*	31.1	31.1	30.7	100.0	71.6	72.6	45.9	50.6
5 **U1KHB2**, *Pseudoalteromonas citrea*	30.8	30.5	30.2	71.6	100.0	74.5	45.1	49.6
6 **A0A5H2Y128**, *Pseudoalteromonas sp. A25*	31.8	31.3	31.0	72.6	74.5	100.0	45.8	49.2
7 **A0A2N8KTW4**, *Paucibacter aquatile*	36.7	36.4	36.1	45.9	45.1	45.8	100.0	54.5
8 **A0A516SCT3**, *Chitinimonas arctica*	33.9	34.0	34.7	50.6	49.6	49.2	54.5	100.0

Figure S3: Percent identity matrix of the closest identified *Pt*TamH homologues.
UniProt accession codes are indicated in **bold** text. Multiple sequence alignment was performed using Clustal Omega.

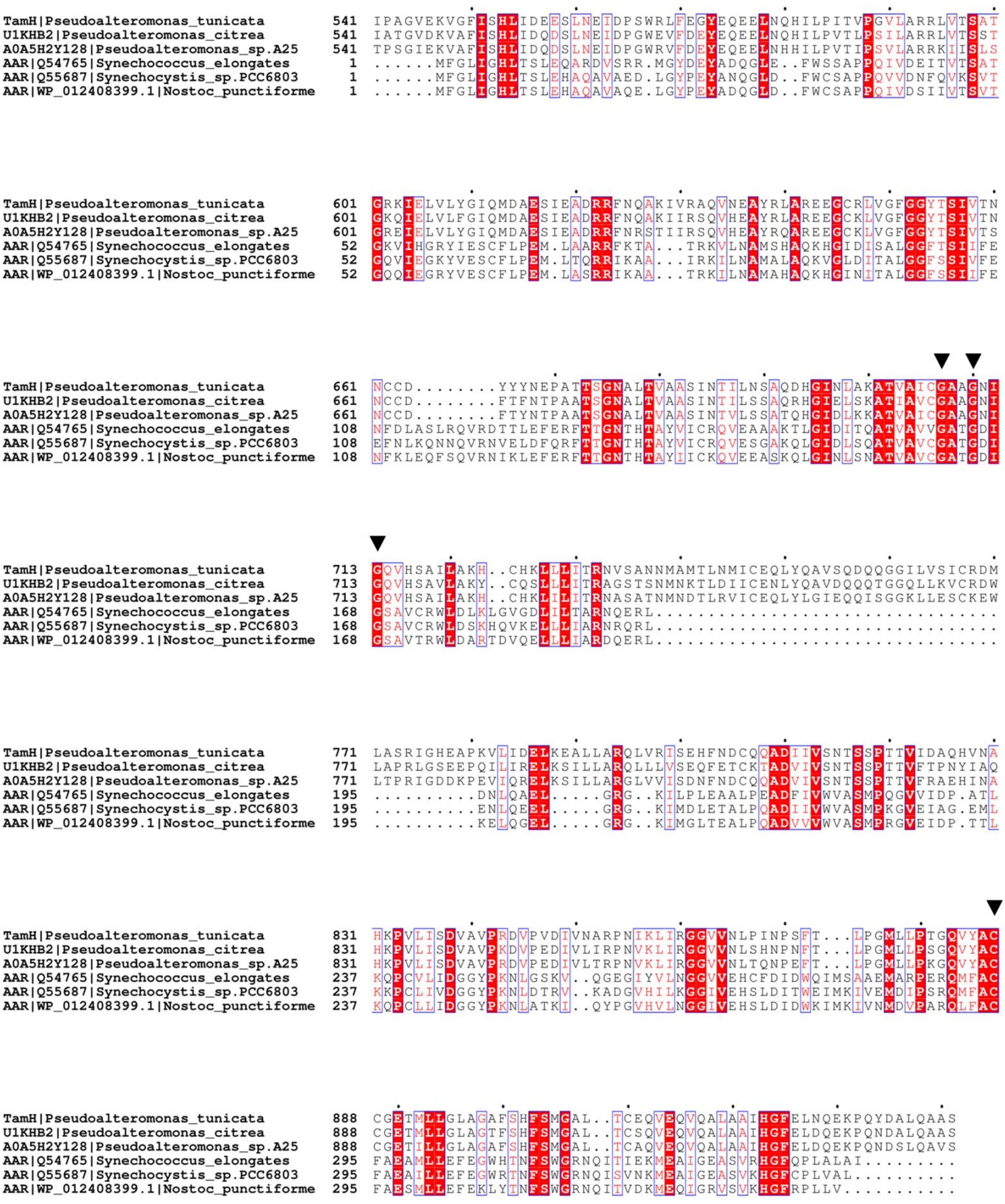

Figure S4: Multiple sequence alignment of *Pt*TamH TR (and known *Pseudoalteromonas* homologues) with cyanobacterial acyl-ACP reductases (AARs).

Putative catalytic/binding residues are indicated with a black triangle (▼).

ATGCAAATTCGTGTTGGACAAGAAGTTTTAAGCCGAGAATCACTGTTAGAAAGTGCAGGTTTATTGGCT

CAATATATCCGTGCGCAAGGCGATATGTTGACTTGGCAAAAAGAGGATGAGCGCATTGAAGTACTGGA

CATGGTCGGTGGCTTTGGCAGTACCTTATTGGGTCATAATCATCCAGAATTGCTTGCAACTATGCAGCA

AAGCCTCAGTAGTTTAAGGCCAATGTGGGTGCAAGGTGCTGAGCGACCTGTAGCAAAACAATTACGCA

ATGCACTTGCGCAAAAGTTGCTACGAGAAACAGGCAAAAAATACAGTATTGTTTTACTCAATACTGGCA

CTGAGGCAGTAGAAGCTGGATTAAAGCATGCGCAATATGAGTTTTTTCAGCGTTTGCAGCACATTCAAC

AACACTGTGATACCAACTGGCGAGAATTTAAATTGCGTCTCGCACGTAATGAAATCCAGCTCACTTCAG

AGTTTTATCTTGAGTGTGAGCGGTTATTACAGCAAGAGCCTATTGAAAGTCTTGAAGAGTTGCAGCGTG

CAGTGCAAATGCGTAATCAAGCCGTGTTTAATTCATCGGGTAAAATAGCCGCATTTAAAGGCGATTTTC

ATGGTAAAACCCGAGGCAGCCTTGCAACGACTTATAATCGCGATGCACGTCTGCCATTTATTGCCAAT

AATCCTGATGCAATATTTATTGATGATGAAGCGCAGTTTAAGGCGACCTTAGCAAGTTGGCAAAAAGCC

TATTTTACCATTGAGTTTGCGCCATTACGTTTGCAGAAAAAGCCTGTAAATTTGTTAACTGCGCTAATTT

ATGAACCCATTCAGGGTGAAGGCGGTGTGCGCCCACTTAATGCGCGTTACTGTGCTTTATTAAACGAG

CTTAAACTCAGTCATCCTGATGTCGCGATTATCGCAGATGAAATTCAATGTGGTTTAGGTCGTACTGGG

CAATTTTTAGAAAGCCAAGCGATAAATACACCCAATGATTATATTACGTTGGCAAAATCACTGGGTGGT

GGACTGTGTAAAATCTCCGCTCTCGCTGTGGAGCAAACTCGTTACCACGAAGAATTTAGCATGCTCCA

TAGCACCACTTTTGCTGATGATGATCTCAGCAGTGCGGTCGCGCTAAAAACACTGGCAATTTTAGAAC

GTGATGAGTTGACCTTAAAAGCCGCACATTTAGGCGAGCAATTTACGACAGCACTCAATGCGTTAGCA

CTTGAATATCCCGATATGATAGCGGATATACGAGGTAAAGGCTGTATGCTTGGCATCGAGCTTGCTGC

CCAAGAGAATCATCCAAGCGCCACAATAGCAGGCTTGGATGAGCAAAATATGCTGGCAATGGCGATTG

CTGGACATTTGTTACATCGTCATCATATTCGTGTATTGCCTTCATTGGGGAAACGTCGAGTATTGCGTT

TGCAACCTTCAGCTTATTTGGATGCCGCAAATATTGCGTTGGTCGTTGACGCACTAAAAGAGACCTTTG

AGCTTATTCGTCATCATCATGTTGCAACTTTGCTTGCGCATCTTATTCATACCGATAAACCACACAGCG

CATTTGCAACGGCTTATCAACATCCAATTCGTGAAGAGGCAATTCCTGCTGGTGTCGAAAAAGTAGGTT

TTATCAGTCACTTAATTGATGAAGAAAGCTTAAATGAAATAGACCCTAGTTGGCGTTTGTTTGAGGGCT

ATGAGCAAGAAGAGCTTAATCAACATATTTTACCTATTACCGTCCCGGGTGTTTTGGCGCGCAGGTTAG

TCACCTCTGCTACCGGTCGTAAAATTGAGCTCGTTTTGTATGGTATTCAAATGGATGCTGAGAGCATTG

AGGCCGACCGTCGCTTTAATCAAGCTAAAATTGTGCGAGCTCAAGTCAACGAAGCGTATCGCTTAGCC

CGTGAAGAAGGCTGCCGTTTAGTTGGATTTGGCGGTTATACCTCAATCGTCACTAATAACTGCTGCGA

CTATTATTACAACGAGCCTGCTACAACGTCAGGTAATGCGCTGACAGTTGCGGCCAGTATTAATACTAT

TTTAAACAGTGCGCAAGATCATGGTATCAACCTCGCAAAAGCAACGGTCGCTATTTGTGGCGCCGCTG

GCAATATCGGCCAAGTACACAGCGCGATATTAGCAAAACATTGCCATAAGCTGCTGTTAATTACTCGTA

ATGTCAGCGCGAATAATATGGCAATGACCTTAAATATGATTTGTGAGCAGTTATATCAAGCAGTGAGCC

AAGATCAACAAGGGGGAATTTTAGTCTCTATCTGTCGAGATATGCTTGCTTCACGCATTGGTCATGAGG

CACCAAAGGTCTTAATCGACGAGCTCAAGGAGGCGTTATTAGCCAGACAATTGGTACGTATTAGCGAA

CATTTTAACGATTGCCAGCAGGCTGACATCATTGTCAGTAATACCAGTAGTCCAACAACGGTTATTGAT

GCGCAACATGTTAATGCGCATAAACCGGTACTGATCAGTGATGTTGCCGTGCCCCGTGATGTGCCAGT

AGATATCGTAAACGCACGTCCGAATATTAAACTTATTCGTGGCGGAGTGGTAAATCTTCCTATTAATCC

GAGCTTTACTCTACCTGGAATGTTATTACCAACAGGTCAGGTTTACGCCTGTTGTGGCGAGACTATGTT

ACTGGGTTTAGCGGGGGCGTTTAGTCATTTTAGTATGGGCGCGTTAACCTGTGAGCAGGTTGAGCAG

GTGCAAGCCTTAGCTGCAATTCATGGATTTGAATTGAATCAGGAGAAGCCGCAATATGACGCCTTGCA

AGCCGCTAGCTGA

Figure S5: Sequence of *Pt*TamH gene.
The sequence of the *Pt*TamH gene (Uniprot: A4C5V8, GenBank: EAR29362)

MSYYHHHHHHDYDIPTTENLYFQGAMEIRVGQEVLSRESLLESAGLLAQYIRAQGDMLTWQKEDERIEVLDMVGGFGSTLLGHNHP

ELLATMQQSLSSLRPMWVQGAERPVAKQLRNALAQKLLRETGKKYSIVLLNTGTEAVEAGLKHAQYEFFQRLQHIQQHCDTNWREF

KLRLARNEIQLTSEFYLECERLLQQEPIESLEELQRAVQMRNQAVFNSSGKIAAFKGDFHGKTRGSLATTYNRDARLPFIANNPDAIFIDD

EAQFKATLASWQKAYFTIEFAPLRLQKKPVNLLTALIYEPIQGEGGVRPLNARYCALLNELKLSHPDVAIIADEIQCGLGRTGQFLESQAI

NTPNDYITLAKSLGGGLCKISALAVEQTRYHEEFSMLHSTTFADDDLSSAVALKTLAILERDELTLKAAHLGEQFTTALNALALEYPDMIA

DIRGKGCMLGIELAAQENHPSATIAGLDEQNMLAMAIAGHLLHRHHIRVLPSLGKRRVLRLQPSAYLDAANIALVVDALKETFELIRHH

HVATLLAHLIHTDKPHSAFATAYQHPIREEAIPAGVEKVGFISHLIDEESLNEIDPSWRLFEGYEQEELNQHILPITVPGVLARRLVTSATG

RKIELVLYGIQMDAESIEADRRFNQAKIVRAQVNEAYRLAREEGCRLVGFGGYTSIVTNNCCDYYYNEPATTSGNALTVAASINTILNSA

QDHGINLAKATVAICGAAGNIGQVHSAILAKHCHKLLLITRNVSANNMAMTLNMICEQLYQAVSQDQQGGILVSICRDMLASRIGHE

APKVLIDELKEALLARQLVRISEHFNDCQQADIIVSNTSSPTTVIDAQHVNAHKPVLISDVAVPRDVPVDIVNARPNIKLIRGGVVNLPIN

PSFTLPGMLLPTGQVYACCGETMLLGLAGAFSHFSMGALTCEQVEQVQALAAIHGFELNQEKPQYDALQAAS 966

Figure S6: *Pt*TamH recombinant protein sequence.
The recombinant protein sequence of the pEHISTEV construct of *Pt*TamH (966 aa) with an N-terminal TEV protease-cleavable HisTag shown in bold.

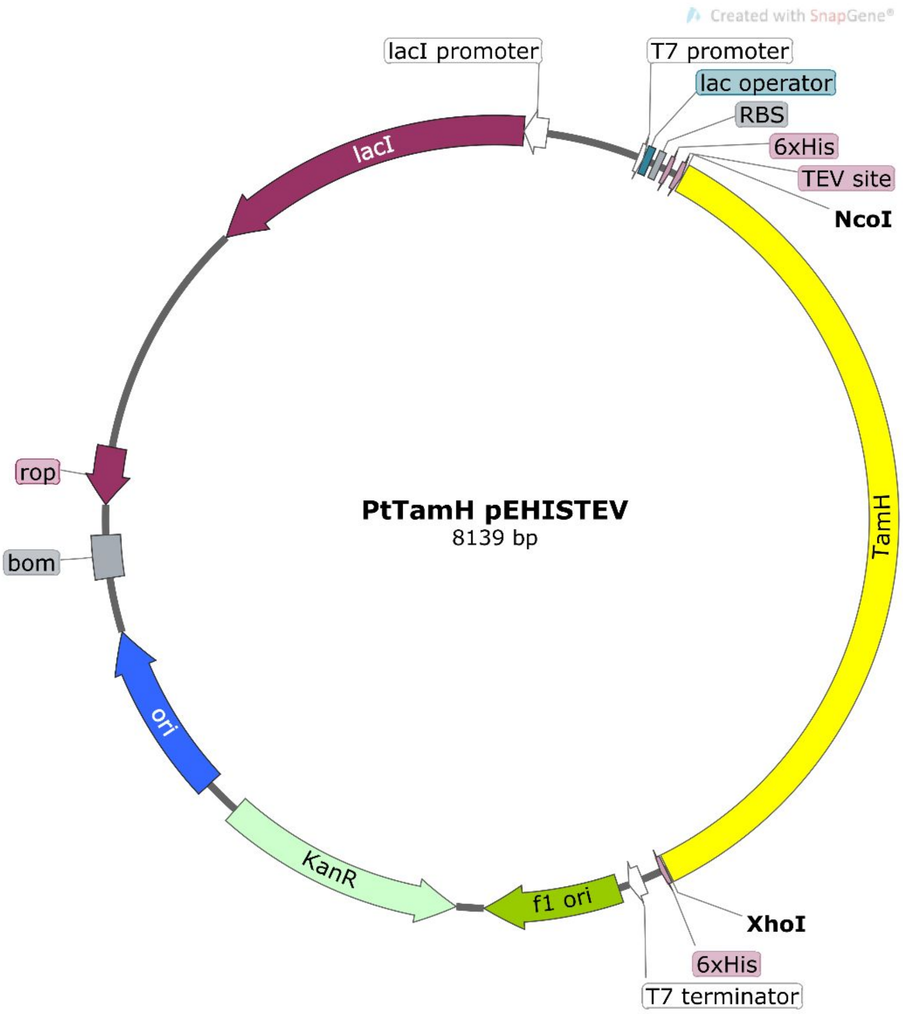

Figure S7: *Pt*TamH pEHISTEV plasmid map.
*Pt*TamH expression plasmid map, cloned into pEHISTEV using the NcoI and XhoI restriction sites.

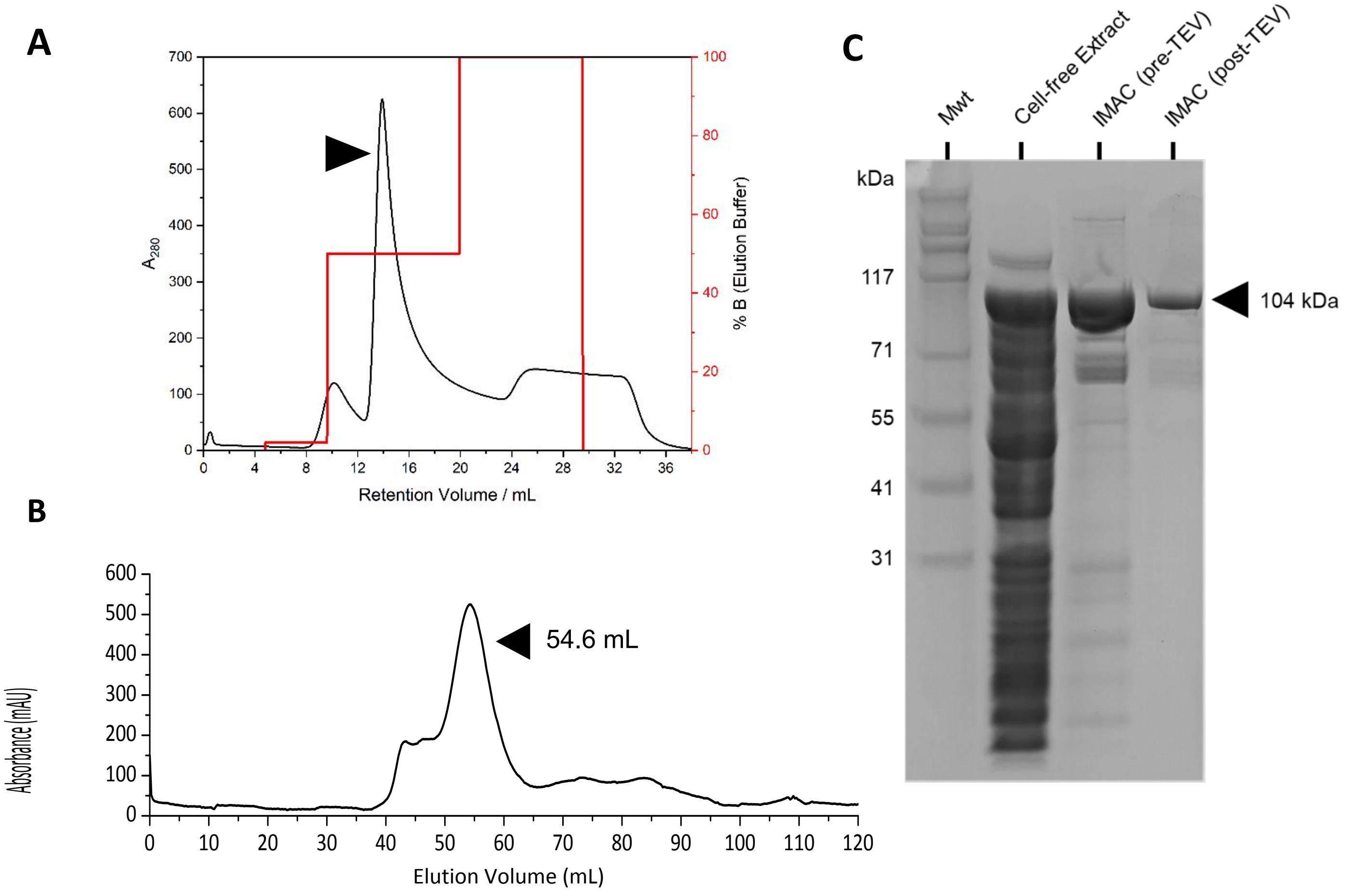

Figure S8: *Pt*TamH purification.

A. IMAC chromatogram showing elution of protein with increasing imidazole as % B buffer increases. **B.** Chromatogram of Superdex S200 GFC monitored at 280 nm showing peak elution at 54.6 mL consistent with a protein dimer and **C.** SDS-PAGE gel of *Pt*TamH purification steps showing the marker (Mwt), cell free extract, and purity after IMAC, pre- and post-TEV cleavage. Initial characterisation consisted of Superdex S200 GFC, but due to high purity after IMAC, *Pt*TamH was used after the 2nd IMAC column.

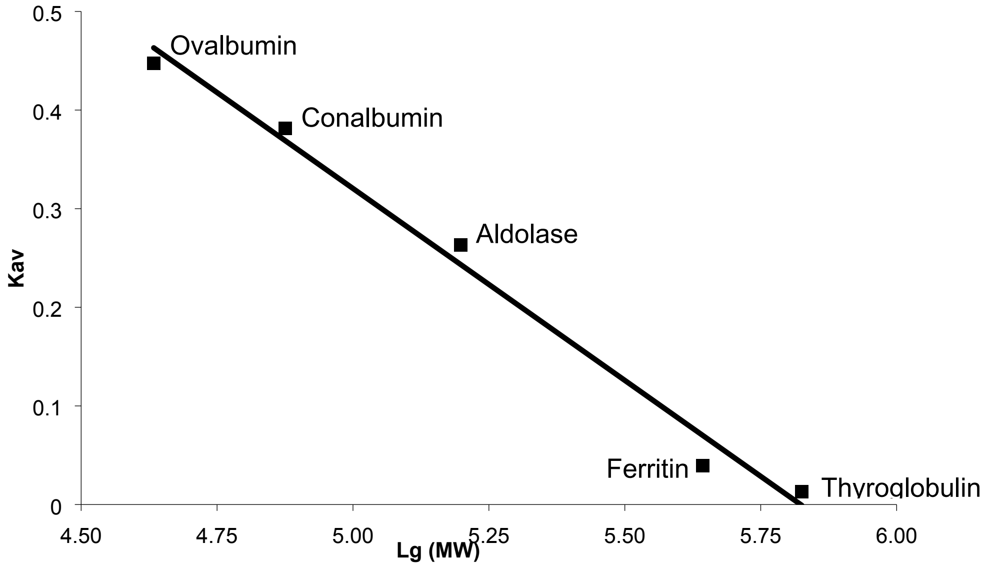

The MW of each protein is estimated using the following:

$$MW = 10^{\frac{K_{av} - 2.2665}{-0.3892}}$$

$$K_{av} = \frac{V_e - V_o}{V_t - V_o}$$

Where:

MW is measured in Da

V_e = Elution volume

V_o = Void volume (44 ml)

V_t = Total bed volume (120 ml)

Figure S9: Superdex HiLoad 16/60 S200 calibration curve.

Calibration curve of the Superdex HiLoad 16/60 S200 size exclusion chromatography column and resulting equation used for the estimation of protein molecular weight (MW).

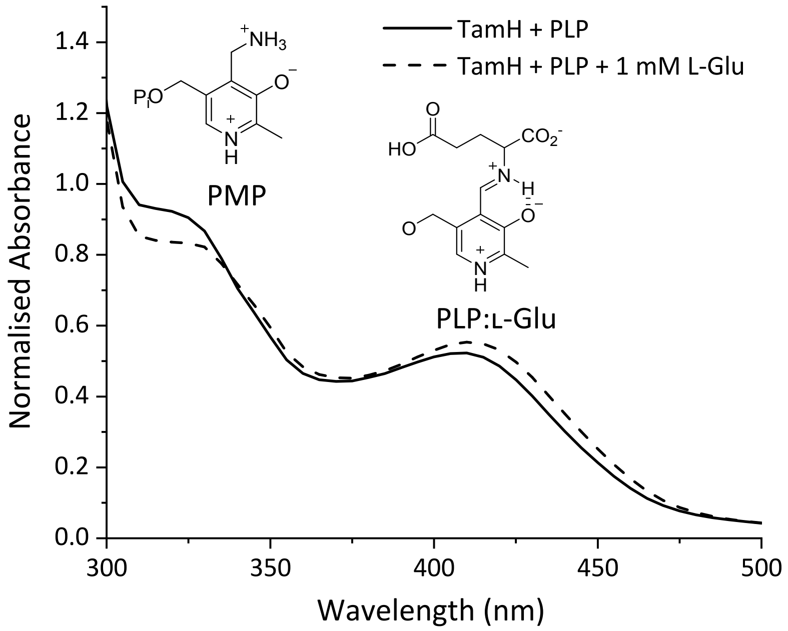

Figure S10: UV-Vis PLP binding spectrum of *Pt*TamH.

UV-Vis scan (300 nm-500 nm) of *Pt*TamH incubated with PLP. Changes in absorbance observed upon addition of amine donor ʟ-Glu (1 mM).

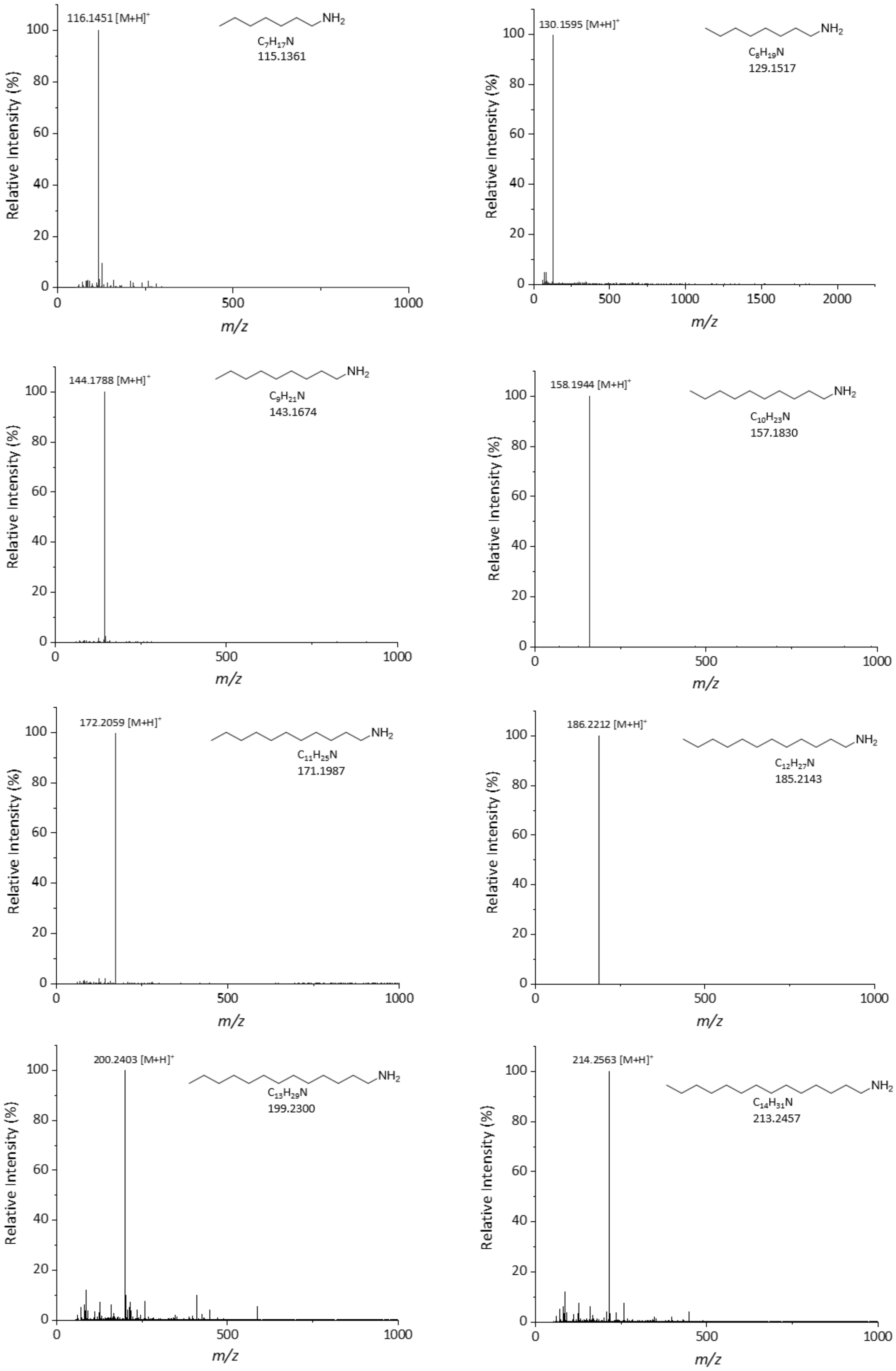

Figure S11: Extracted ion chromatograms (EICs) of C_7-C_{14} amine products after incubation with *Pt*TamH.
LC-ESI MS confirmation of the formation of the C_7-C_{14} aldehyde with *Pt*TamH TA domain in the presence of ʟ -Glu in triplicate. These EICs are representative each reaction tested.

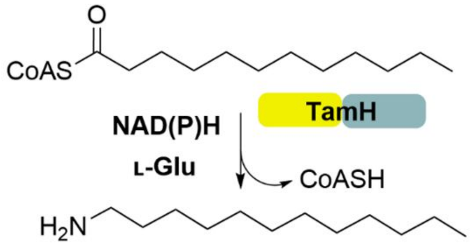

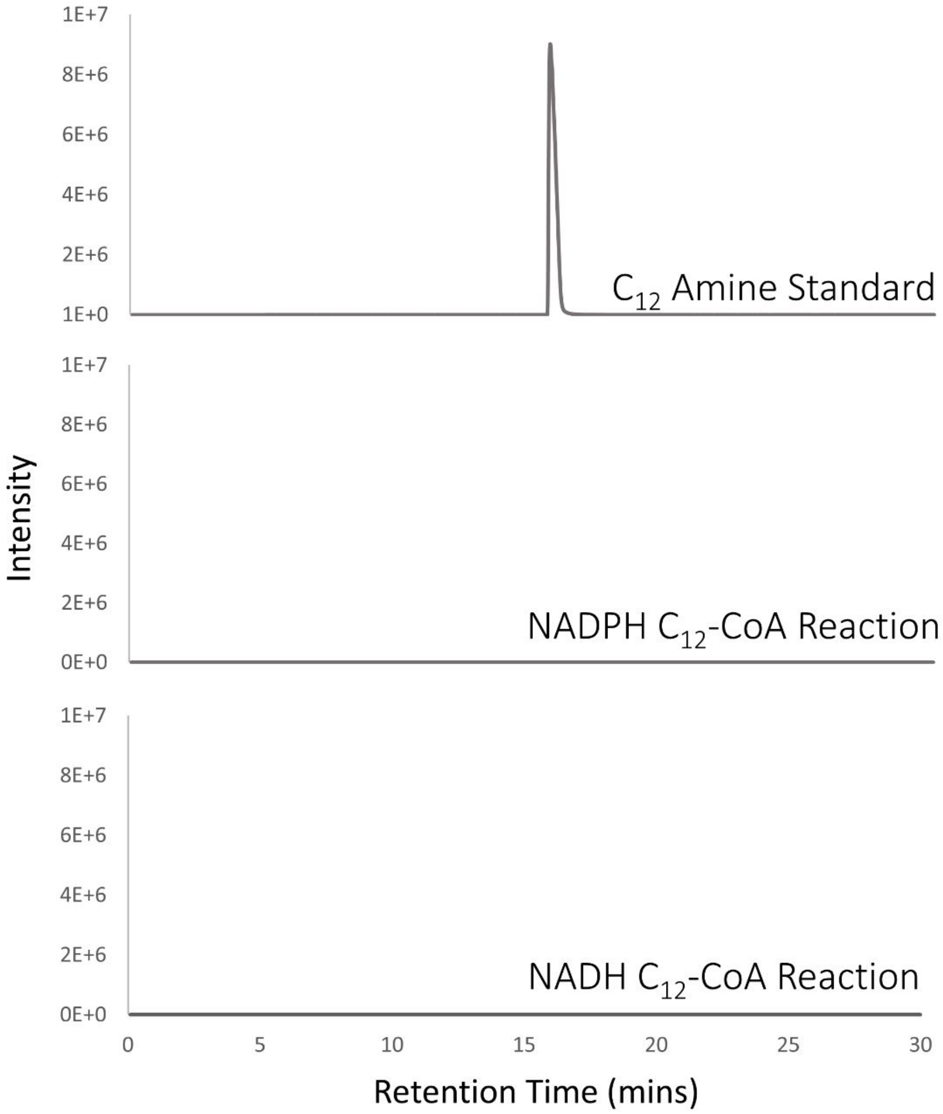

Figure S12: Attempted *Pt*TamH + C_{12}-CoA reaction for the formation of the C_{12} amine product.
EICs for the C_{12} amine standard ion with an *m/z* = 186.2222 Da which corresponds that predicted for the C_{12} primary amine ($[M+H]^+$, $C_{12}H_{28}N$), along with the reactions of *Pt*TamH incubated with either NADH or NADPH each of which results in no product formation.

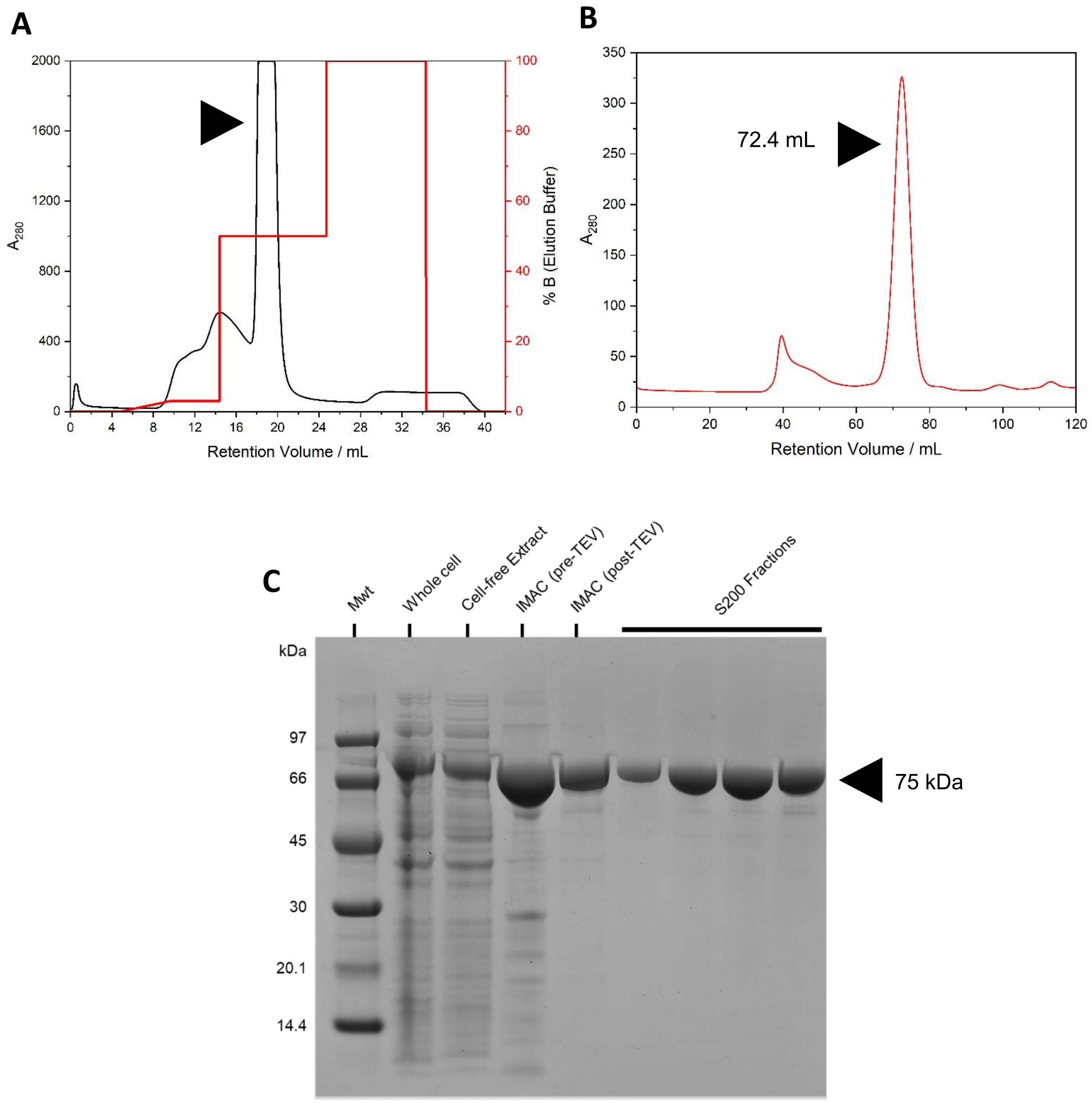

Figure S13: *Pt*TamA purification.

A. IMAC chromatogram showing elution of protein with increasing imidazole as % B buffer increases. **B.** Chromatogram of Superdex S200 GFC monitored at 280 nm showing peak elution at 72.4 mL and **C.** SDS-PAGE gel of *Pt*TamA purification steps showing the marker (Mwt), whole cell, cell free extract, and purity after IMAC, pre- and post-TEV cleavage and S200 fractions.

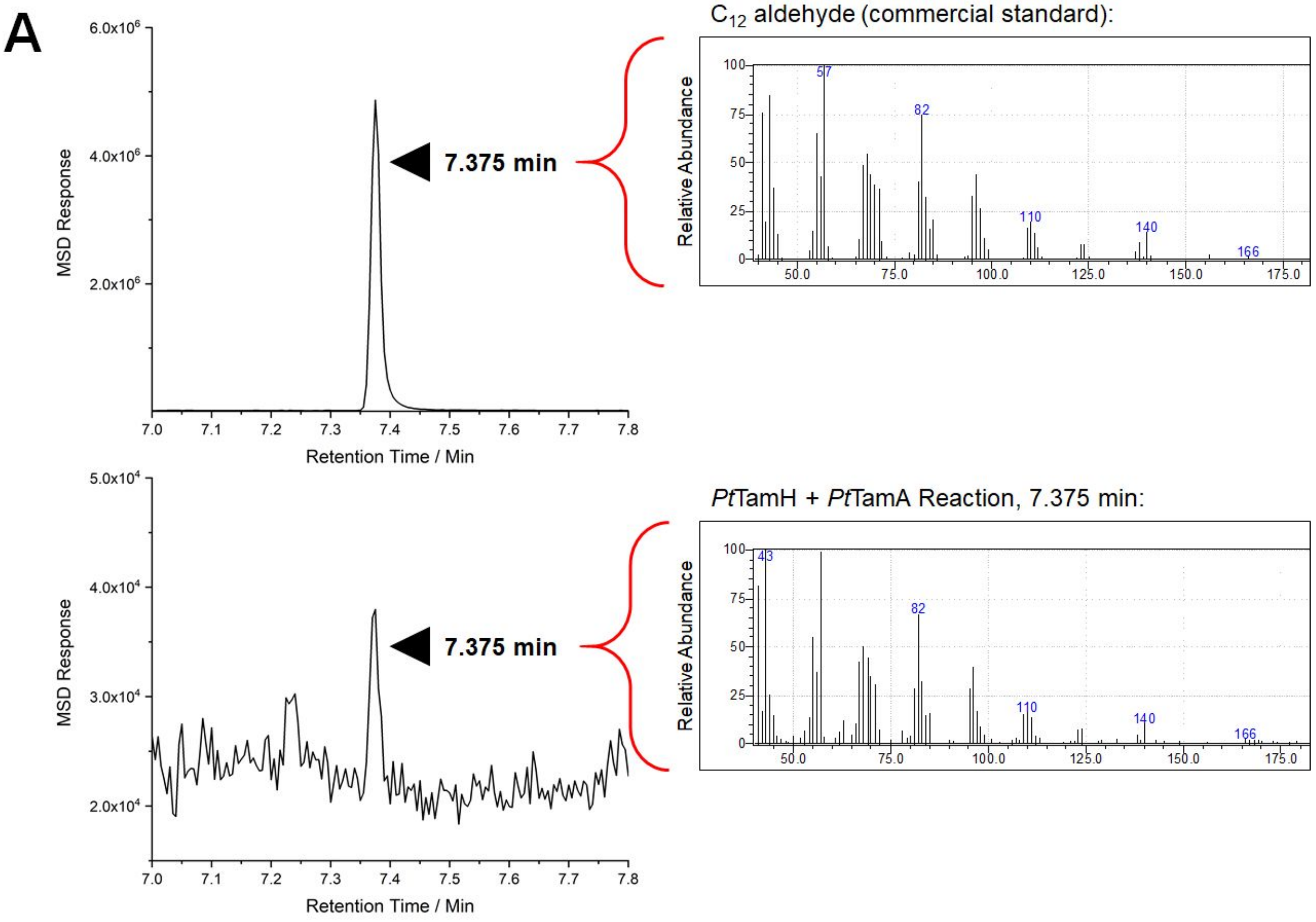

B

Observed Ion	Observed m/z	Inference
M-141	43	Loss of $^{\bullet}C_8H_{16}CHO$
M-140	44	McLafferty
M-127	57	Loss of $^{\bullet}C_7H_{14}CHO$
M-44	140	Loss of CH_2=CH-OH
M-18	166	Loss of H_2O

Figure S14: Detection of C_{12} aldehyde from a *Pt*TamH + *Pt*TamA cascade lacking ʟ-Glu.

A. Aligned, representative GC-MS chromatograms of a C_{12} aldehyde commercial standard (top) and an organic extraction of the *Pt*TamA + *Pt*TamH biocatalytic cascade (bottom). The EI-MS fragmentation is displayed to the right of each peak. **B.** Assignment of some diagnostic fragment ions characteristic of C_{12} aldehyde.

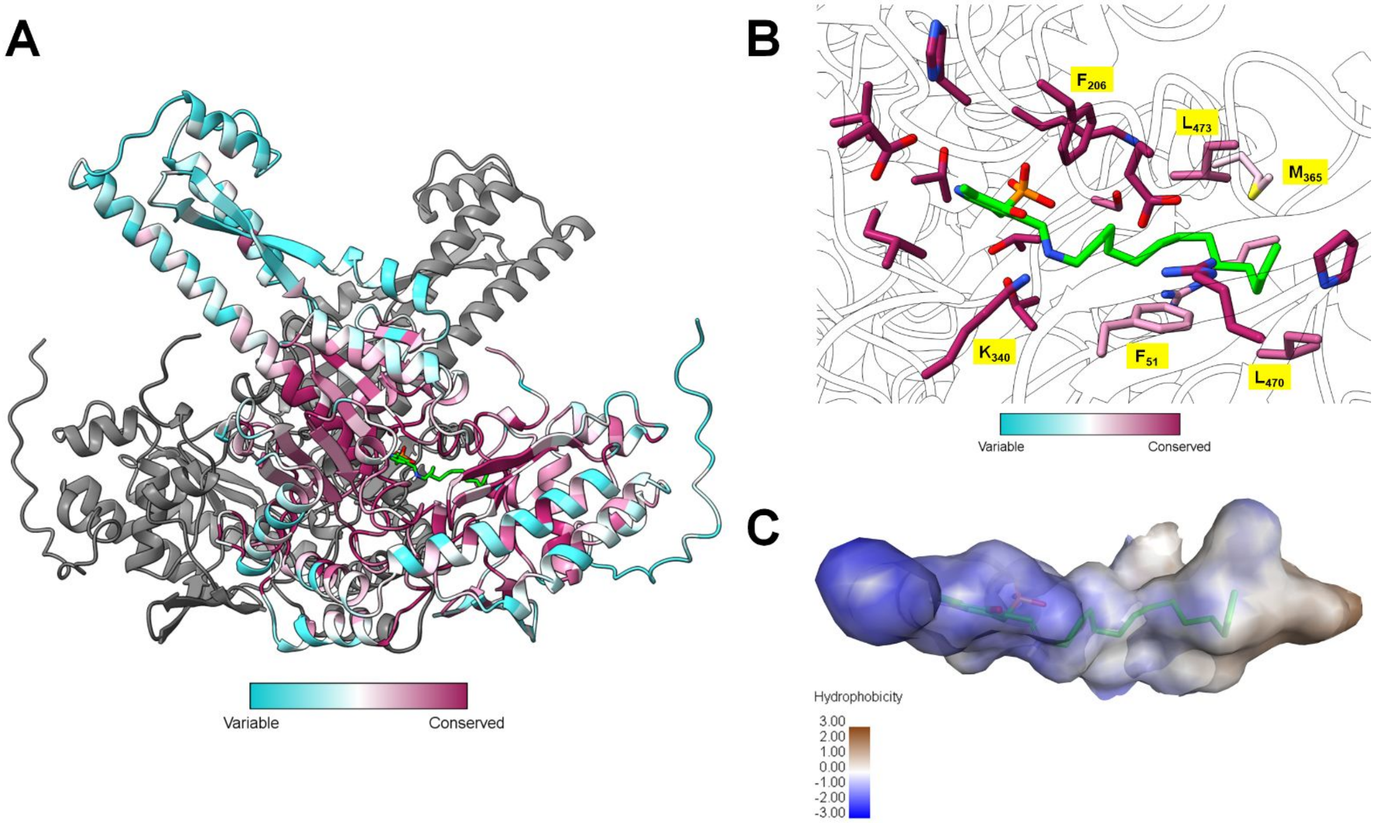

Figure S15: *Pt*TamH ω-TA conservation analysis and ligand docking.
The docked C_{12} external aldimine is coloured lime green. **A.** ConSurf[12-15] conservation scores mapped onto the ω-TA domain. **B.** The docked C_{12} external aldimine stabilized by several conserved residues. **C.** Kyte-Doolittle hydropathy surface of the predicted binding pocket enclosing the docked C_{12} external aldimine.

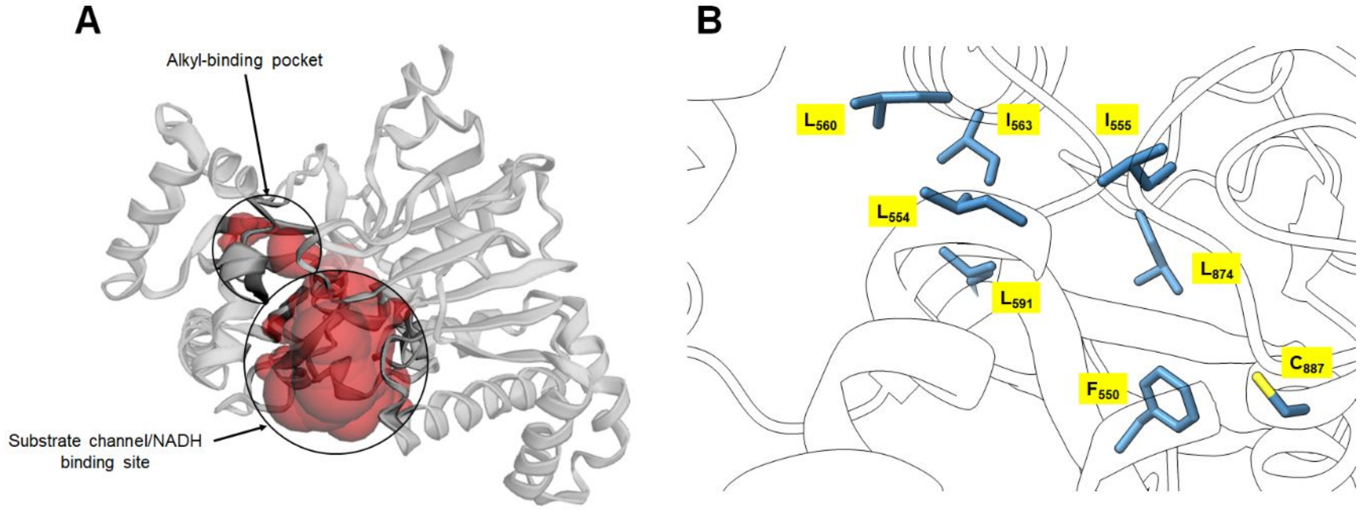

Figure S16: *Pt*TamH TR topological analysis.
A. A pocket with a surface area and volume of 827.0 Å^2 and 712.9 Å^3 was identified by CASTp 3.0. The function of each pocket region was inferred by structural homology to *Se*AAR (PDB: 6JZY). **B.** Residues comprising a putative hydrophobic alkyl-binding pocket adjacent to the catalytic C_{887}.

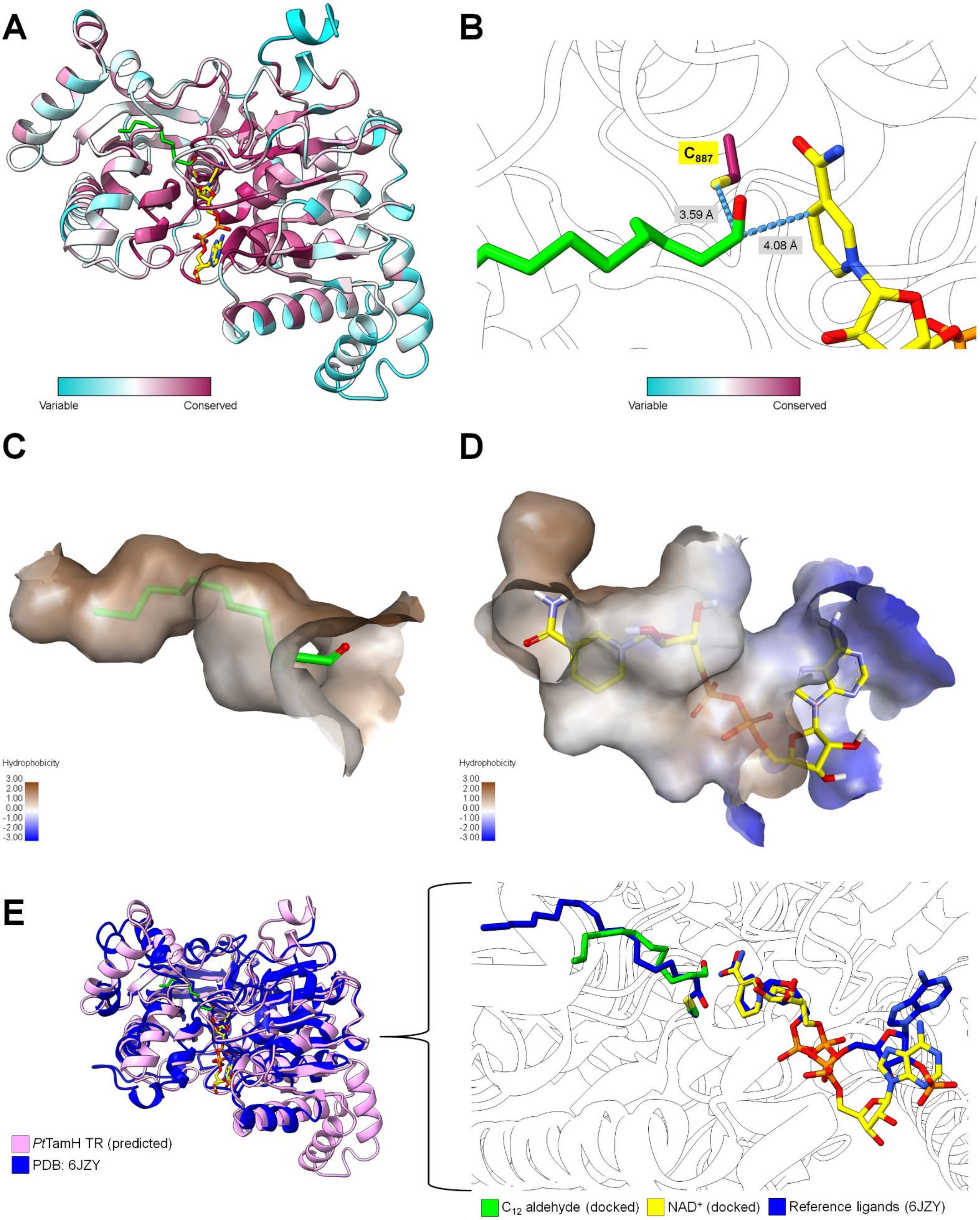

Figure S17: *Pt*TamH TR conservation analysis and ligand docking.
The docked C_{12} aldehyde and NAD^+ are coloured lime green and yellow respectively. **A.** ConSurf[12-15] conservation scores mapped onto the TR domain. **B.** The C_{12} aldehyde and NAD^+ docked in close proximity to the putative catalytic C_{887}. **C.** Kyte-Doolittle hydropathy surface of the predicted binding pocket enclosing docked C_{12} aldehyde. **D.** Kyte-Doolittle hydropathy surface of the predicted NAD^+ binding pocket. **E.** The *Se*AAR crystal structure (PDB: 6JZY) superimposed on the predicted *Pt*TamH TR. The *Se*AAR NADPH and stearoyl chain are shown in blue.

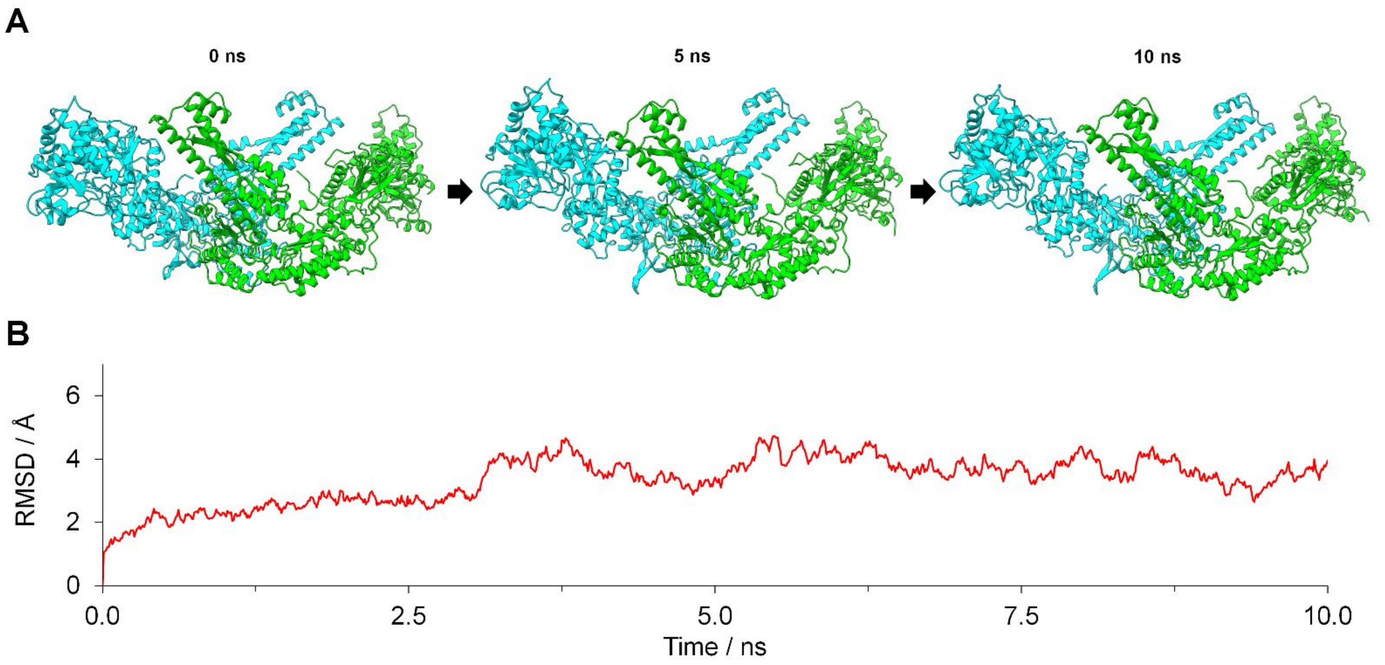

Figure S18: Structural morphology of the *Pt*TamH complex during MDS.
A. *Pt*TamH trajectory frames sampled at the start, middle and endpoint of the MDS. **B.** 1D RMSD plot (vs. t = 0 ns) of the simulated *Pt*TamH complex.

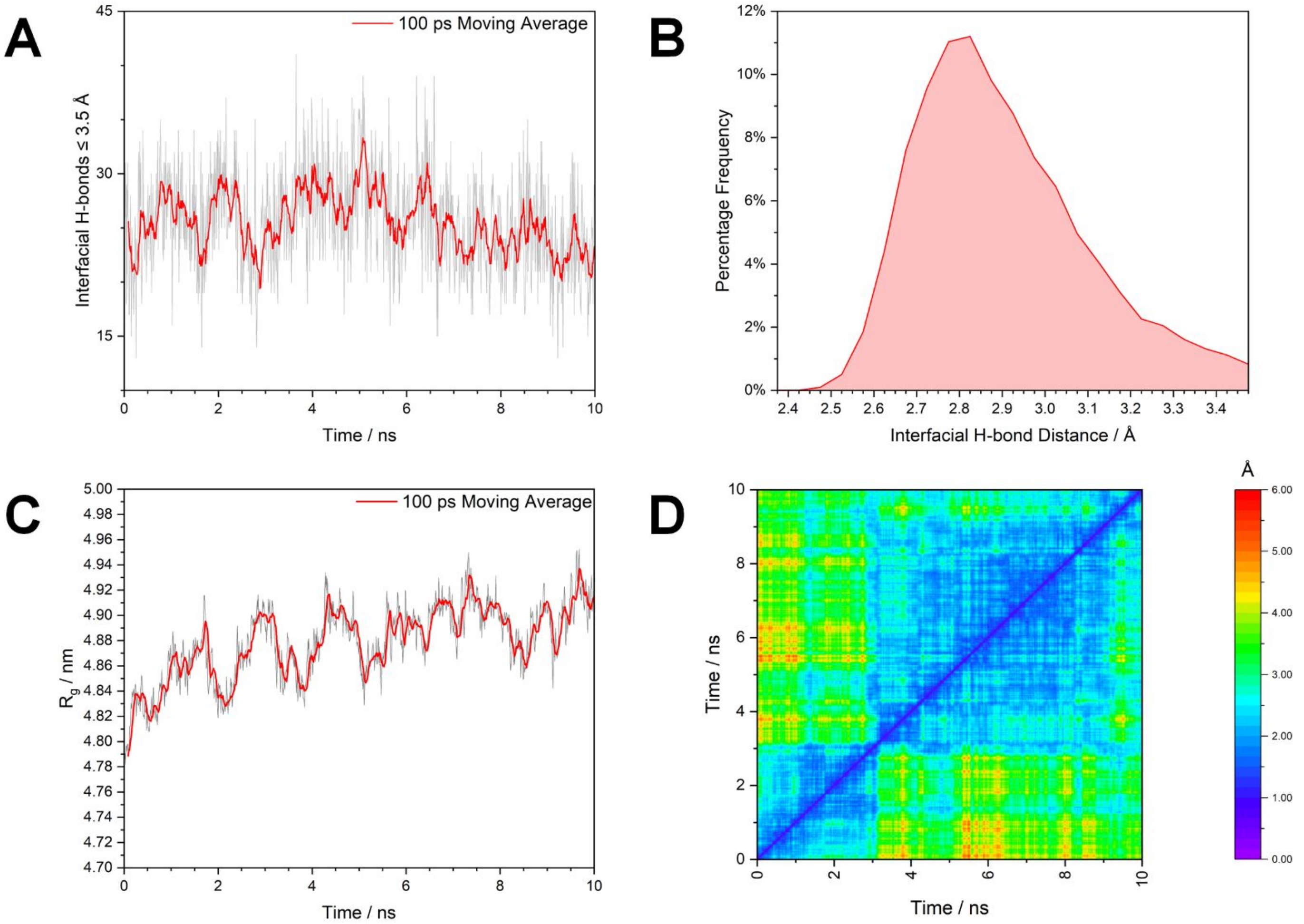

Figure S19: A summary of the *Pt*TamH MDS.
A. An examination of the number of inter-chain hydrogen bonds over time. **B.** A distance distribution of the inter-chain hydrogen-bonded contacts. **C.** The radius of gyration (R_g) of the *Pt*TamH complex over time. **D.** A pairwise (2D) RMSD map computed between structures at every time point of the simulation.

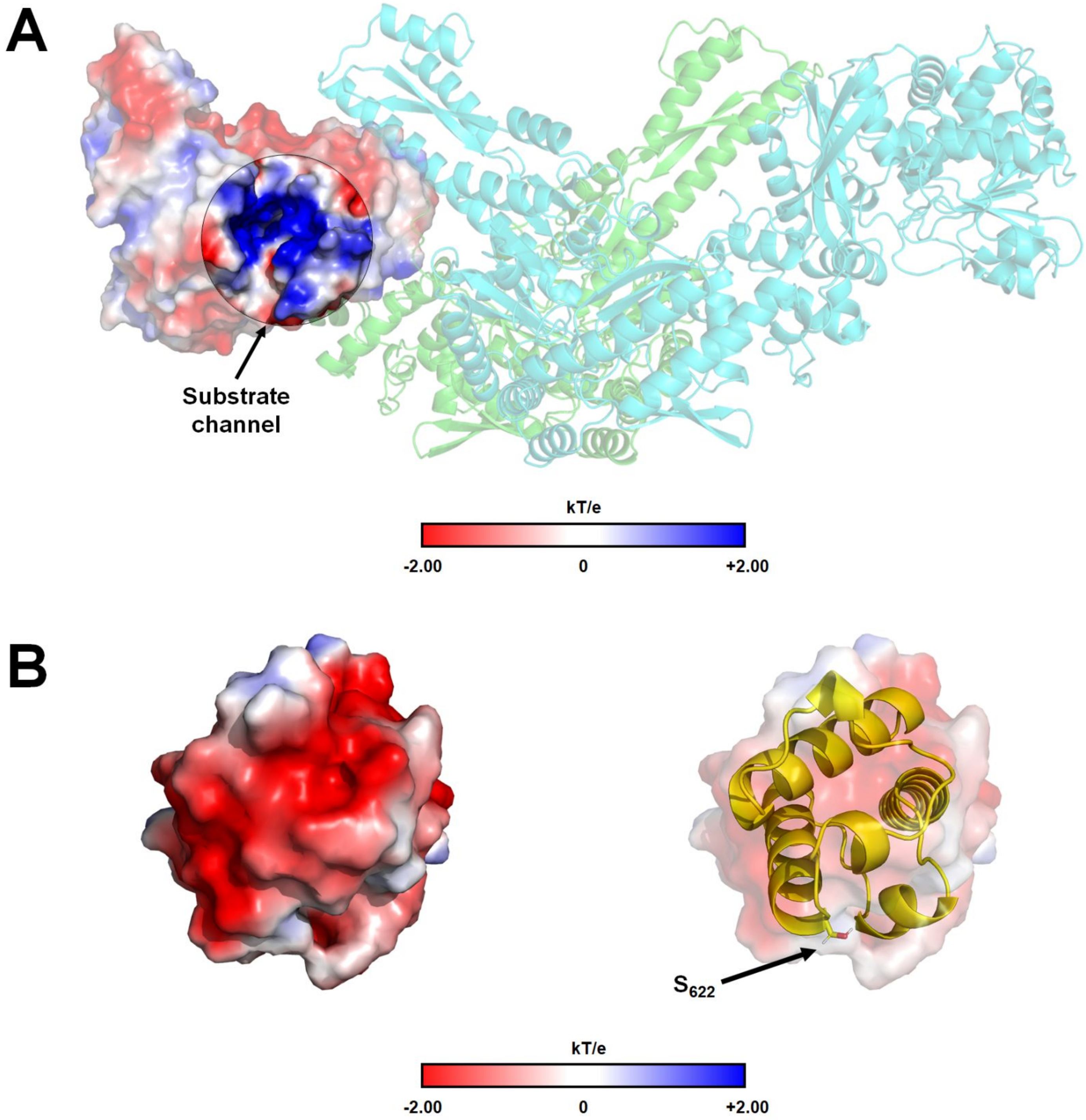

Figure S20: APBS electrostatics analysis of *Pt*TamH and the *Pt*TamA ACP domain.

A. The electrostatic potential surface of *Pt*TamH TR highlighting the entrance to the substrate channel. **B.** The electrostatic potential surface of *Pt*TamA ACP highlighting the position of the critical S_{622} on the electronegative surface of the protein.

References:

1. Liu, H.; Naismith, J. H., A simple and efficient expression and purification system using two newly constructed vectors. *Protein Expression Purif.* **2009,** *63*, 102-111.
2. Prasad, S.; Khadatare, P. B.; Roy, I., Effect of chemical chaperones in improving the solubility of recombinant proteins in Escherichia coli. *Appl. Environ. Microbiol.* **2011,** *77*, 4603-9.
3. Pettersen, E. F.; Goddard, T. D.; Huang, C. C.; Meng, E. C.; Couch, G. S.; Croll, T. I.; Morris, J. H.; Ferrin, T. E., UCSF ChimeraX: Structure visualization for researchers, educators, and developers. *Protein Sci.* **2021,** *30*, 70-82.
4. Jurrus, E.; Engel, D.; Star, K.; Monson, K.; Brandi, J.; Felberg, L. E.; Brookes, D. H.; Wilson, L.; Chen, J.; Liles, K.; Chun, M.; Li, P.; Gohara, D. W.; Dolinsky, T.; Konecny, R.; Koes, D. R.; Nielsen, J. E.; Head-Gordon, T.; Geng, W.; Krasny, R.; Wei, G.-W.; Holst, M. J.; McCammon, J. A.; Baker, N. A., Improvements to the APBS biomolecular solvation software suite. *Protein Sci.* **2018,** *27*, 112-128.
5. Tian, W.; Chen, C.; Lei, X.; Zhao, J.; Liang, J., CASTp 3.0: computed atlas of surface topography of proteins. *Nucleic Acids Res.* **2018,** *46*, W363-W367.
6. Morris, G. M.; Huey, R.; Lindstrom, W.; Sanner, M. F.; Belew, R. K.; Goodsell, D. S.; Olson, A. J., AutoDock4 and AutoDockTools4: Automated docking with selective receptor flexibility. *J. Comput. Chem.* **2009,** *30*, 2785-2791.
7. Eberhardt, J.; Santos-Martins, D.; Tillack, A. F.; Forli, S., AutoDock Vina 1.2.0: New Docking Methods, Expanded Force Field, and Python Bindings. *J. Chem. Inf. Model.* **2021,** *61*, 3891-3898.
8. Trott, O.; Olson, A. J., AutoDock Vina: Improving the speed and accuracy of docking with a new scoring function, efficient optimization, and multithreading. *J. Comput. Chem.* **2010,** *31*, 455-461.
9. Abraham, M. J.; Murtola, T.; Schulz, R.; Páll, S.; Smith, J. C.; Hess, B.; Lindahl, E., GROMACS: High performance molecular simulations through multi-level parallelism from laptops to supercomputers. *SoftwareX* **2015,** *1-2*, 19-25.
10. Huang, J.; MacKerell Jr, A. D., CHARMM36 all-atom additive protein force field: Validation based on comparison to NMR data. *J. Comput. Chem.* **2013,** *34*, 2135-2145.
11. Pettersen, E. F.; Goddard, T. D.; Huang, C. C.; Couch, G. S.; Greenblatt, D. M.; Meng, E. C.; Ferrin, T. E., UCSF Chimera--a visualization system for exploratory research and analysis. *J. Comput. Chem* **2004,** *25* (13), 1605-12.
12. Ashkenazy, H.; Erez, E.; Martz, E.; Pupko, T.; Ben-Tal, N., ConSurf 2010: calculating evolutionary conservation in sequence and structure of proteins and nucleic acids. *Nucleic Acids Res.* **2010,** *38*, W529-W533.
13. Celniker, G.; Nimrod, G.; Ashkenazy, H.; Glaser, F.; Martz, E.; Mayrose, I.; Pupko, T.; Ben-Tal, N., ConSurf: Using Evolutionary Data to Raise Testable Hypotheses about Protein Function. *Isr. J. Chem.* **2013,** *53* (3-4), 199-206.
14. Glaser, F.; Pupko, T.; Paz, I.; Bell, R. E.; Bechor-Shental, D.; Martz, E.; Ben-Tal, N., ConSurf: identification of functional regions in proteins by surface-mapping of phylogenetic information. *Bioinformatics,* **2003,** *19*, 163-4.
15. Landau, M.; Mayrose, I.; Rosenberg, Y.; Glaser, F.; Martz, E.; Pupko, T.; Ben-Tal, N., ConSurf 2005: the projection of evolutionary conservation scores of residues on protein structures. *Nucleic Acids Res.* **2005,** *33*, W299-302.
